# Nanoscale-Structured Hybrid Bragg Stacks with Orientation- and Composition-Dependent Mechanical and Thermal Transport Properties: Implications for Nacre Mimetics and Heat Management Applications

**DOI:** 10.1021/acsanm.2c00061

**Published:** 2022-03-02

**Authors:** Theresa Dörres, Malgorzata Bartkiewicz, Kai Herrmann, Marius Schöttle, Daniel Wagner, Zuyuan Wang, Olli Ikkala, Markus Retsch, George Fytas, Josef Breu

**Affiliations:** ‡ Bavarian Polymer Institute (BPI) and Department of Chemistry, University of Bayreuth, Universitätsstrasse 30, Bayreuth 95440, Germany; § Max Planck Institute for Polymer Research, Ackermannweg 10, Mainz 55128, Germany; # School of Mechanical and Electrical Engineering, University of Electronic Science and Technology of China, Chengdu, Sichuan 611731, China; ¶ Department of Applied Physics, Aalto University, P.O. Box 15100, Espoo FI-00076, Finland

**Keywords:** organic−inorganic nanocomposites, Brillouin light spectroscopy, thermal conductivity, mechanical tensor, nacre mimetic

## Abstract

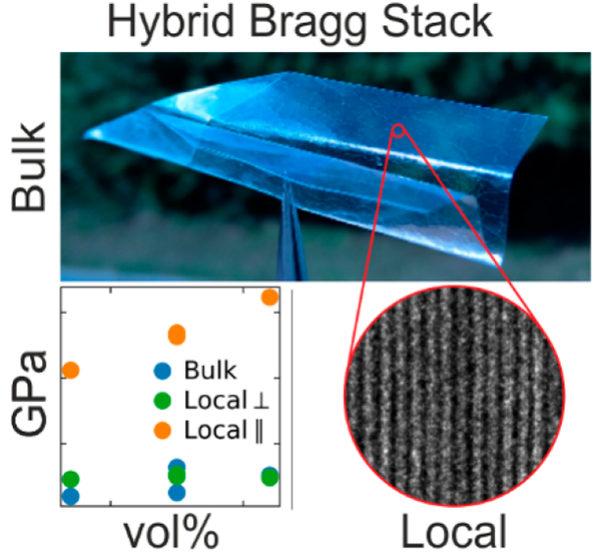

Layered nanomaterials fascinate researchers for their mechanical, barrier, optical, and transport properties. Nacre is a biological example thereof, combining excellent mechanical properties by aligned submicron inorganic platelets and nanoscale proteinic interlayers. Mimicking nacre with advanced nanosheets requires ultraconfined organic layers aimed at nacre-like high reinforcement fractions. We describe inorganic/polymer hybrid Bragg stacks with one or two fluorohectorite clay layers alternating with one or two poly(ethylene glycol) layers. As indicated by X-ray diffraction, perfect one-dimensional crystallinity allows for homogeneous single-phase materials with up to a 84% clay volume fraction. Brillouin light spectroscopy allows the exploration of ultimate mechanical moduli without disturbance by flaws, suggesting an unprecedentedly high Young’s modulus of 162 GPa along the aligned clays, indicating almost ideal reinforcement under these conditions. Importantly, low heat conductivity is observed across films, κ_⊥_ = 0.11–0.15 W m^–1^ K^–1^, with a high anisotropy of κ_∥_/κ_⊥_ = 28–33. The macroscopic mechanical properties show ductile-to-brittle change with an increase in the clay volume fraction from 54% to 70%. Conceptually, this work reveals the ultimate elastic and thermal properties of aligned layered clay nanocomposites in flaw-tolerant conditions.

## Introduction

Biological composites have attracted growing interest because they are concomitantly strong and tough and have low density.^1−3^ While strong materials resist plastic deformations, tough materials are resistant to catastrophic fracture. A classic strong and tough biological composite is pearl of nacre, which consists of a high volume fraction (95 vol %) of aligned reinforcing inorganic aragonite platelets (Young’s modulus *E* = 70 GPa, thickness 200–900 nm, and lateral sizes 5–8 μm) and a soft biopolymer layer (thickness 10–50 nm), which are hierarchically arranged in a so-called “brick-and-mortar” structure.^2,4,5^ In spite of extensive research, the reasons for the extraordinary mechanical properties are not fully understood because of the complex combination of several mechanisms, hampering rational designs of advanced bioinspired materials. Grossly oversimplified, it has been postulated that the ceramic component provides reinforcement, whereas the biopolymer enables the distribution of localized stresses and provides fracture energy dissipation. However, the actual behavior is much more complex and also involved mineral bridges between the reinforcements.^5^

What is significant in nacre is the considerable thickness of ca. 10–50 nm of the macromolecular layers, which, in turn, requires a large thickness of the reinforcing platelets, while assuring a high volume fraction of reinforcements. It has been conceived that, in this way, the dynamic polymer chains can promote the fracture energy dissipative transformations. Two opposite approaches can be considered for nacre-mimetic designs: (1) If thick submicron inorganic layers are really needed to concomitantly allow for a high filler content while keeping the thick polymer layers, they have to be biomineralized *in situ*; otherwise, excessive packing defects remain. A high volume fraction cannot be achieved using prefabricated, thick, solid, inorganic sheets. (2) By contrast, if nanometric sheets that are strong in tension but flexible in bending are used, their packing defects can be largely relieved, but a high reinforcement volume fraction cannot be easily achieved unless the polymer layers are ultrathin. Both approaches have been adopted toward nacre-inspired materials, ranging from *in situ* mineralization to the use of various nanosheets.^6−20^ The latter approach has been widely exploited because it may allow stronger reinforcements than those when relatively brittle ceramics are used. Nanosheets can also serve other functions, such as improving the gas barrier properties^21−24^ or incorporating electrical conductivity by using, for example, mXene.^25^

Although approaches have been provided to allow a high ultimate strength in nacre-mimetic materials^20^ and some approaches have shown even nacre-like toughness in notched fracture mechanical studies,^18,26,27^ composite elastic moduli have typically remained much smaller than expected from the constituents. For example, the nacre-mimetic materials based on clay typically show Young’s modulus (*E*) values of some tens of gigapascal (GPa), while pure clay nanosheets show *E* values of 146–171 GPa.^28,29^ Even complex combinations of graphene modifications, clay, and carbon nanotubes show only a modest *E* modulus value of 130 GPa,^20^ signifying a fundamental problem of transferring the reinforcement elastic properties to the composite system. The question remains as to whether the low modulus value is due to insufficient stress transfer across the interfaces or due to defects. A second question relates to using nanometric two-dimensional (2D) nanosheets, of which several types have been used, such as clays, graphene and its modifications, mXenes, and titanates: Could a high volume fraction of reinforcements still be achieved in cases that use an organic “matrix”? Also, what are the mechanical properties, in particular the elastic moduli, of the composite systems, considering the ultraconfinement of the polymer layers? In addition, if such properties are achieved, does the presence of elastic anisotropy indicate the presence of thermal anisotropy? Finally, how much does defect tolerance affect the macroscopic mechanical properties?

Herein we address these questions by constructing one-dimensional (1D) crystalline arrangements (“hybrid Bragg stacks”) of nanosheets and polymer layers with a superior translational periodicity. We use synthetic sodium fluorohectorite ([Na_0.5_]^inter^[Mg_2.5_Li_0.5_]^oct^[Si_4_]^tet^O_10_F_2_), which delaminates based on the rare phenomenon of repulsive osmotic swelling, yielding an unprecedented thickness homogeneity and complete separation.^30,31^ This contrasts the standard liquid-phase exfoliation of classically used natural clays, such as montmorillonite, which only provides a broad distribution of thicknesses and poorly defined materials. Importantly, osmotic swelling produces a nematic liquid-crystalline phase that can be homogeneously mixed with a polymer.

Regarding selection of the polymer, so far only two polymers, poly(vinylpyrrolidone) (PVP)^21,32,33^ and poly(ethylene glycol) (PEG),^34−36^ have been discovered to allow for the formation of single-phase hybrid Bragg stacks with a discrete composition. This is contrasted by poly(vinyl alcohol),^16,18,22^ ethoxylated poly(ethyleneimine)/poly(acrylic acid),^23^ and sodium carboxymethylcellulose,^37^ which undergo (partial) phase separation because of insufficient interaction of the polymer with the clay surface or interlayer cation.

Osmotic swelling of fluorohectorite leads to single (Hec) or double (DS) nanosheets, allowing for the controlled fabrication of nanosheets that are either 1 or 2 nm thick.^38^ Dispersions with appropriately adjusted volume fractions of fluorohectorite and PEG can be spray-coated to generate hybrid Bragg stacks. Thereby, PEG can be intercalated as a single macromolecule or stacked macromolecules between these two filler morphologies, resulting in four types of 1D crystalline, transparent hybrid Bragg stack monodomain films (i.e., Hec/2PEG, Hec/1PEG, DS/2PEG, and DS/1PEG), with the filler content spanning from 54 to 82 vol % in discrete, material-inherent (“quantized”) compositions. This allows one for the first time to combine a superior periodicity with four distinct filler contents. In combination with its large-scale homogeneity and transparency, it allows for an unprecedented mechanical and thermal analysis. As a noncontact and nondestructive technique for measuring the elastic moduli at zero strain and a submicron resolution, Brillouin light spectroscopy (BLS) provides unique access to the full elastic tensor of anisotropic materials.^32,39^ The present single-phase, transparent, and, to some degree, tunable periodic structures enable rich information to be extracted from the BLS spectra. These requirements, however, are not satisfied by the classic montmorillonite-filled clay-based nacre mimetics. Insights from BLS can be complemented with thermal transport properties that in view of the nanometer spacing correlate with the speed of sound from the BLS experiments and also probe the fundamental properties and anisotropy of the hybrid Bragg stacks.

## Results

### Fabrication of 1D Crystalline, Quantized Hybrid Bragg Stacks

Among the 2D materials, only a handful of compounds are known to show repulsive osmotic swelling^40^ to allow for gentle delamination into nanosheets of uniform thickness while preserving the equivalent diameter of the pristine crystals. Synthetic [Na_0.5_]^inter^[Mg_2.5_Li_0.5_]^oct^[Si_4_]^tet^O_10_F_2_, obtained by melt synthesis,^41^ followed by long-term annealing,^38^ shows such a rare phenomenon. Additionally, it possesses phase purity, homogeneous charge density, and thus uniform intracrystalline reactivity. Because of its exceptional charge homogeneity, the as-synthesized fluorohectorite can be transformed into an ordered interstratification by partial ion exchange with NH_4_^+^. Na^+^ and NH_4_^+^ ions segregate to different interlayers, and the two types of interlayers strictly alternate in this ordered heterostructure, which represents a thermodynamically stable state. Because of the lower hydration enthalpy of NH_4_^+^, hydration in this interlayer type is completely blocked.

When the pristine fluorohectorite is immersed in deionized water, individual 1-nm-thick nanosheets are obtained.^38^ This suspension is not isotropic but represents a nematic phase, where the nanosheets are held in a cofacial arrangement because of their electrostatic repulsion. When the heterostructure (selective Na^+^ exchange to NH_4_^+^ in every other interlayer) is immersed in water, every other interlayer type is blocked from osmotic swelling, and it, consequently, spontaneously delaminates into 2-nm-thick nanosheets (DS),^29^ where two hectorite layers are connected via a nonswelling interlayer of NH_4_^+^ ions. To obtain the most frequently used montmorillonite platelet diameter (typically less than 300 nm), ultrasonication is applied to reduce the equivalent diameters of Hec and DS to 340 and 406 nm, respectively (Figure S1). In nacre, the macromolecules in the organic layers are particularly soft;^5^ therefore, PEG with a low glass transition temperature (*T*_g_ = −20 °C) was selected. Being water-soluble and nonionic, it does not interfere with electrostatic stabilization of the nematic suspensions. Moreover, PEG is known to form complexes with Na^+^,^42^ and, consequently, intercalated phases with two different PEG volumes may be obtained^36^ because phase separation is counterpoised by interaction with the interlayer cation.

**Figure 1:**
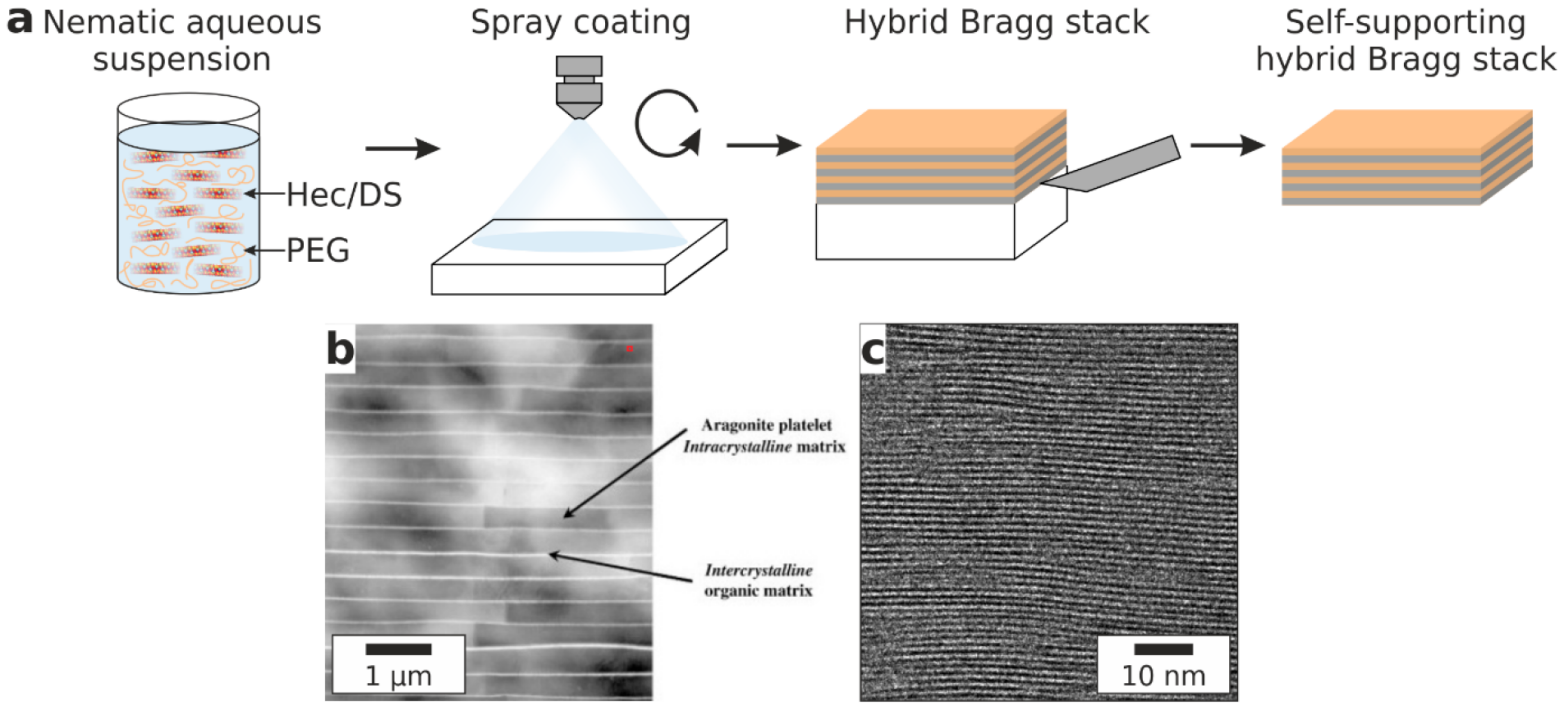
(a) Preparation of the free-standing hybrid Bragg stacks. Nematic aqueous suspensions are repeatedly spray-coated and can be peeled off in order to achieve free-standing films. (b) Typical TEM image of the microarchitecture of sheet nacre.^5^ The red square corresponds to the dimension of part c. Reprinted with permission from Stempflé, P.; Pantalé, O.; Rousseau, M.; Lopez, E.; Bourrat, X. Mechanical properties of the elemental nanocomponents of nacre structure. *Mater. Sci. Eng. C***2010**, *30* (5), 715. Copyright 2010 Elsevier. (c) Comparison to the TEM image of an exemplary hybrid Bragg stack highlighting the vastly different length scales.

Independent of the nanosheet lateral sizes and thicknesses, dilute (<1 vol %) suspensions represent nematic phases in water, with the nanosheets separated by large distances (>50 nm).^31^ Therefore, solution compounding could be achieved simply by mixing the nematic suspensions with different amounts of an aqueous PEG solution, which preserves the nematic character of the suspension, as is evidenced by small-angle X-ray scattering (SAXS; Figure S2). Spray coating onto poly(ethylene terephthalate) foils, followed by gentle drying, produced transparent hybrid films (Figure 1a). The nanosheets align parallel to the substrate because of their large aspect ratio and form a monocrystalline film, with the stacking direction oriented perpendicular to the foil. Transmission electron microscopy (TEM) images corroborate the nice periodic homogeneity of the sample films. Such films are self-supporting and can be easily peeled off from the substrate. A comparison of the characteristic length scales to illustrate the difference between a typical nacre and the hybrid Bragg stacks produced here is shown in Figure 1b,c.

**Figure 2:**
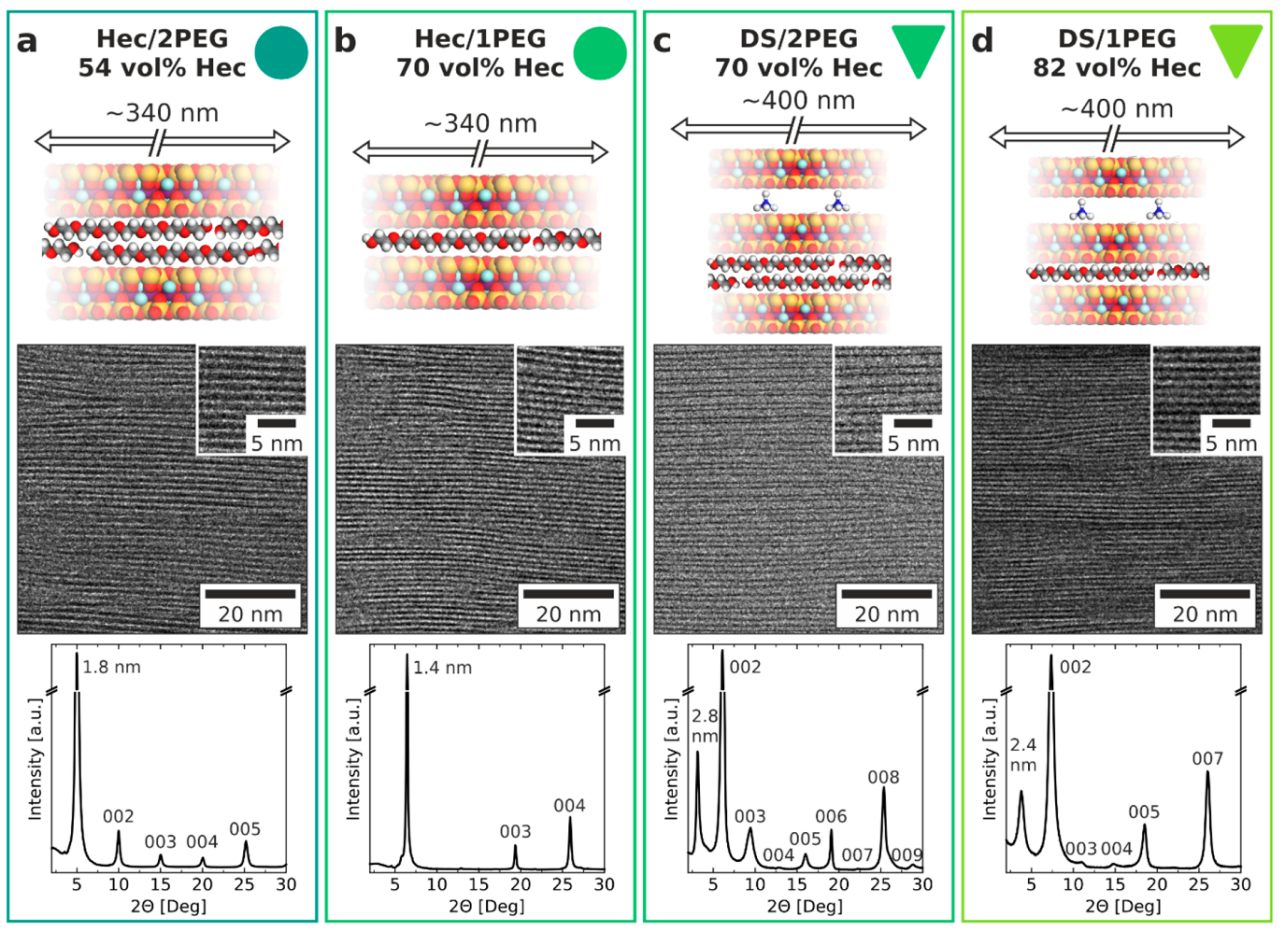
Hybrid clay nacre mimetics with quantized composition. Schematic structures of the hybrid Bragg stacks (top). TEM images (middle) of argon-milled slices confirming the superior order and long-range periodicity. The dark lines are single Hec sheets. The XRD patterns confirm the nearly perfect 1D crystallinity, as indicated by the intense basal reflections, rational 00*l* series, and long-range periodicity. The volume fraction of clay increases from left to right with (a) hectorite with 2PEG, (b) hectorite with 1PEG, (c) DS with 2PEG, and (d) DS with 1PEG.

Previous studies have reported intercalated phases of layered silicates with PEG volume contents of 46 vol %^34−36,43,44^ and 30 vol %^36^ corresponding to periodicities of 1.8 and 1.4 nm, respectively. The present observed periodicities (1.77 and 1.38 nm for Hec/2PEG and Hec/1PEG, respectively; Figure 2, bottom) agree well with the published values. The PEG volume contents were iteratively optimized to yield these superior periodicities. According to Meuring’s rule,^45^ the quality of the 1D crystallinity is exhibited by a small full width at half-maximum (fwhm). Our samples with optimized PEG contents feature intense basal reflections of a rational 00*l* series of up to the fifth and fourth order and a very low coefficient of variation (CV) of the 00*l* series (0.3–0.6% for Hec/2PEG and Hec/1PEG, respectively; Figure 2, bottom, and Table S1). As expected, the fwhm minima (Figure S3) at 54 vol % (Hec/2PEG) and 70 vol % (Hec/1PEG) of Hec coincide with the CV minima (Figure S3). Such low CVs imply extraordinarily uniform interlayer heights, which usually require ionic or molecular interlayer cations of the same type and well-defined size and shape. Observing such a low CV for a hybrid Bragg stack with intercalated macromolecules is quite surprising because even low-molecular-weight polymers are intrinsically polydisperse and thus are expected to adopt different conformations.

Similar to the Hec-based films, the periodicity and filler content of the films could be varied systematically at discrete steps by applying the DS filler without compromising the homogeneity or quality of the 1D crystallinity. As described for Hec/2PEG and Hec/1PEG, the PEG content was carefully optimized, and the optimum 1D crystallinity was observed for 70 and 82 vol % of DS fillers with CV values of 1.6 and 1.0 % for DS/2PEG and DS/1PEG, respectively (Figure 2, bottom). The DS/1PEG hybrid exhibits a record filler content of 82 vol %. As expected, the *d* spacings increase by 1 nm upon going from Hec to DS for DS/2PEG and DS/1PEG (2.79 and 2.38 nm, respectively), corresponding to the thickness of a hectorite nanosheet because NH_4_^+^ fits into the surface corrugations and does not require additional space. The quality of the 1D crystallinity of the DS Bragg stacks is comparable to that of the Hec Bragg stacks, as indicated by the intense reflections, and a rational 00*l* series visible up to the ninth and seventh order for DS/2PEG and DS/1PEG, respectively (Figure 2, bottom). Moreover, the TEM images of both DS hybrids also showed an exceptional long-range order (Figure 2, middle). The compositions of Hec/1PEG and DS/2PEG differ only by the additional NH_4_^+^ interlayer in the latter, which shows up by slight variations in the weight percent content (Figure S4), while the volume content is the same within experimental errors (Table S1). As shown by the X-ray diffraction (XRD) patterns and TEM images, the periodicity of DS/2PEG is doubled, and thus the interface density between PEG and the clay is halved. We use these four distinct, transparent samples with ultimate structural control, a maximum filler content, and polymer confinement to test the intrinsic, direction-dependent thermal and mechanical properties. We include in our analysis an alternative polymer, PVP, which is also capable of forming distinct, crystalline hybrid Bragg stacks with 31 and 40 vol % of Hec.^32^

### Anisotropic Elastic Moduli

BLS has important advantages compared to macroscopic mechanical experiments: (i) measurement of the elastic moduli under zero strain, utilizing thermal phonons in materials, (ii) determination of the elastic tensor components, probing direction-dependent phonon propagation in anisotropic materials, and (iii) insensitivity to material defects due to the submicron resolution of the BLS technique, ensuring determination of the ultimate material elasticity, which should be the ultimate goal of defect engineering. BLS results from the inelastic scattering of light by the longitudinal and transverse phonons propagating in the medium with a wave vector **q**. The magnitude, *q*, depends on the incident laser wavelength, λ = 532 nm, the scattering angle, θ, and the material refractive index, *n* (section S3). Hence, the probed phonon wavelength varies in the range of about 200–2000 nm.

For elastically isotropic media, the BLS spectrum is Doppler shifted at *f* = ±*cq*/2π around the central Rayleigh line, with *c* being the longitudinal (*c*_L_) or transverse (*c*_T_) sound velocity that determines the corresponding longitudinal, , shear, , and Young’s, *E* = 2*G*(1 + *v*) moduli, with ρ being the density and  Poisson’s ratio. Because of the high frequencies of the probed phonons, for all materials at temperatures in the range of *T*_g_ < *T* < *T*_g_ + 100 K, where *T*_g_ is the glass transition temperature, the moduli are truly elastic (i.e., frequency independent).^46^

**Figure 3:**
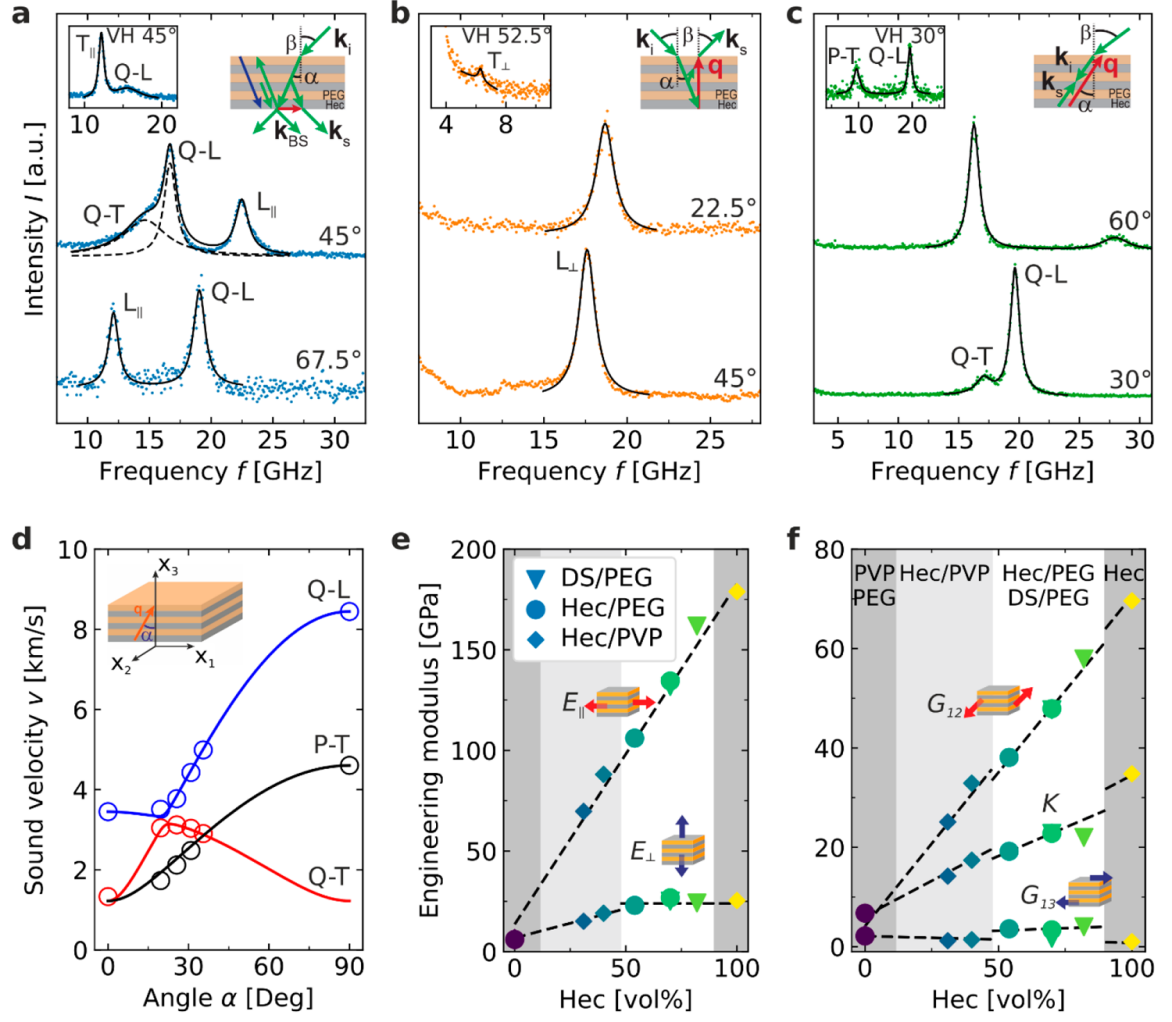
Orientation-dependent elasticity. BLS spectra of the Hec/1PEG sample (with the Lorentzian fits appearing as black lines) recorded at the (a) transmission, (b) reflection, and (c) backscattering geometries in polarized (main panels) and depolarized (left insets) configurations. The solid lines denote the sum of the individual Lorentzians (dotted lines). The right insets in parts a–c show schematics of the transmission, reflection, and backscattering geometries, respectively, where β denotes the incident angle of the laser beam, α is the directional angle between **q**, labeled by red arrows, and the normal to the sample film. **k**_i_, **k**_s_, and **k**_BS_ are the wave vectors of the incident, scattered, and backscattered light inside the sample, respectively. The blue arrow in the right inset to part a indicates the wave vector in the artificial backscattering geometry. The assignment of the peaks in the spectra is explained in the text. (d) Direction-dependent sound velocities of the observed acoustic phonons in the BLS spectra (circles) and from the theoretical representation (solid lines) of Hec/1PEG. (e and f) Composition dependence of the engineering moduli of the hybrid samples (Hec/PEG, circles; DS/PEG, triangles) and pure PEG sample (circles at 0% of the Hec content). Parts e and f also contain the results for the Hec/PVP system (diamonds).^32^ The data included for this system correspond to PVP (0%), double layer of PVP (31 vol % Hec), single layer of PVP (40 vol % Hec), and pure Hec (100%). The schematics in parts e and f visualize the physical meanings of the corresponding moduli.

For elastically anisotropic materials like the hybrid Bragg stacks in this work, determination of the stiffness constants of the elastic tensor requires access to the direction-dependent polarized and depolarized BLS spectra. The methodology has recently been reported for several hard^32^ and soft materials (section S4).^46−48^ However, for an unambiguous determination of the elastic constants, full utilization of the BLS geometries and polarizations is needed. We performed measurements at three different scattering geometries—transmission, reflection, and backscattering—to probe phonon propagation along the directions parallel, normal, and oblique to the present Hec/PEG films, respectively (insets to Figure 3a–c and section S3). Polarization of the incident and scattered light was selected to be either vertical (V) or horizontal (H). Different polarization configurations (e.g., VV and VH) allow for selective examination of the existing modes (section S4).

Figure 3a shows exemplary VV and VH (left inset) BLS spectra recorded in the transmission geometry (right inset) at different laser incident angles, β. In the case of the VV and VH transmission geometries, the spectra should respectively display the in-plane L_∥_ and T_∥_ modes. However, we also observe additional modes (i.e., quasi-longitudinal Q–L and quasi-transverse Q–T) from backscattering contributions due to the internal reflection of the laser beam on the sample’s backside (right inset to Figure 3a).^32,49,50^Figure 3b displays VV and VH (left inset) BLS spectra recorded at the reflection geometry (right inset) at different β angles. Only a single out-of-plane longitudinal mode (L_⊥_) and a single out-of-plane transverse mode (T_⊥_) are present in the VV and VH spectra, respectively. Note that the latter VH spectrum is much weaker than the corresponding in-plane mode (inset to Figure 3a). Figure 3c presents typical VV and HV (left inset) BLS spectra obtained at the backscattering geometry (right inset) at different β angles. The two peaks in the VV spectrum are assigned to Q–T and Q–L modes, whereas the HV spectrum displays an anticipated P–T mode. The second Q–L is due to the polarization scrambling (VV). Parts a–c of Figure 3 refer to the Hec/1PEG sample, while the spectra for the other samples are shown in Figure S6. It is noteworthy that the three different scattering geometries and different polarization configurations allow for complete characterization of the transversely anisotropic material.

After representation of the spectral peaks by Lorentzian lines (solid lines in Figure 3a–c) to yield the frequencies of the observed modes, we computed the direction-dependent sound velocities, *c* = 2π*f*/*q* (section S3). We started from the transmission geometry, for which the magnitude of *q*_∥_ is independent of the refractive index *n*. The dispersion relation *f*(*q*_∥_) for both L_∥_ and T_∥_ is linear (Figure S7) and, hence, *c*(α = 90°) for both phonons is *q*-independent (see section S3 for details). To determine *q*_⊥_ at the reflection geometry and *q*_bs_ at the backscattering geometry, the refractive index of the sample has to be known. We have thus recorded the dispersion *f*(*q*_⊥_), which was forced to be linear through the origin (Figure S7), to determine the value of *n*. In this way, we obtained the sound velocities *c*(α = 0°) for both out-of-plane modes (L_⊥_ and T_⊥_). For the oblique *q* directions in the backscattering measurements (Figure 3c) of a transversely isotropic material, we took into account only those **q** vectors that lie in one plane containing the axis of symmetry. The direction is then defined by the angle α between **q** and the normal to the sample film, in the range of 0–90°; this angle, inside the film (inset to Figure 3a), cannot exceed 50° (Figure 3d) because of the material refractive index (Snell’s law).

For a transversely isotropic material, the elastic stiffness tensor contains five independent elastic constants, which we determined through χ^2^ fitting (section S4).^32^ Because of the relationship between the elasticity tensor components and sound velocities (eqs S1–S7), a theoretical framework of the direction-dependent sound velocities was obtained. The sound velocities of the Q–L, Q–T, and P–T modes are shown in Figure 3d as a function of the angle α in the case of the Hec/1PEG sample along with their theoretical representations (solid lines). Similar results were obtained for the other samples, as shown in Figure S8. The reflection and transmission measurements yield *c*_Q–L_ and *c*_Q–T_ at α = 0° and *c*_Q–L_ and *c*_P–T_ at α = 90°, respectively. Note that the backscattering measurements address all three modes (Q–L, Q–T, and P–T) at intermediate α angles, which are limited below about 50° by the sample’s refractive index.

The theoretical representation of the sound velocities also allowed for designation of the engineering moduli (Figure 3e,f). These are the in-plane Young’s modulus (*E*_∥_), cross-plane Young’s modulus (*E*_⊥_), sliding shear modulus (*G*_13_), torsional shear modulus (*G*_12_), bulk modulus (*K*), and two characteristic Poisson’s ratios (*v*_31_ and *v*_12_, not shown in Figure 3).^51^ For simplicity, we denote the in-plane Young’s modulus as *E*_∥_ (and not as *E*_11_ or *E*_22_) and the out-of-plane Young’s modulus as *E*_⊥_ (and not *E*_33_). The exact values of the elastic stiffness constants and engineering moduli are summarized in Tables S3 and 1, respectively. The results are based on the fitting with two free parameters. The uncertainties are computed according to the principles of uncertainty propagation.

**Table 1:** Engineering Mechanical Moduli of the Hybrid Bragg Stack Films Based on BLS Analysis^a^

sample ID	Hec (vol %)	*E*_∥_ (GPa)	*E*_⊥_ (GPa)	*E*_∥_/*E*_⊥_	*G*_13_ = *G*_23_ (GPa)	*G*_12_ (GPa)	ν_31_ = ν_32_	ν_12_	*K* (GPa)
Hec/2PEG	54	106.1 ± 2.7	23.0 ± 0.5	4.6 ± 0.3	3.6 ± 0.5	38.1 ± 1.5	0.02 ± 0.01	0.39 ± 0.03	19.2 ± 0.7
Hec/1PEG	70	134.5 ± 2.3	26.8 ± 0.5	5.0 ± 0.2	3.4 ± 0.1	47.9 ± 1.2	0.01 ± 0.01	0.40 ± 0.02	22.6 ± 0.8
DS/2PEG	70	132.0 ± 3.0	25.2 ± 0.5	5.2 ± 0.2	1.6 ± 0.6	47.7 ± 1.6	0.03 ± 0.01	0.38 ± 0.02	22.7 ± 0.7
DS/1PEG	82	161.8 ± 3.3	24.2 ± 0.6	6.7 ± 0.2	4.0 ± 0.2	57.9 ± 1.8	0.02 ± 0.01	0.40 ± 0.02	22.0 ± 0.8

a Definitions: *E*_∥_, in-plane Young’s modulus; *E*_⊥_, cross-plane Young’s modulus; *G*_13_ and *G*_23_, sliding shear moduli; *G*_12_, torsional shear modulus; ν_31_, ν_32_, and ν_12_, Poisson’s ratios (ν_*ij*_ represents the strain response in the *j* direction due to a strain in the *i* direction); *K*, bulk (compression) modulus.

Depending on the direction, a systematic variation of the elastic moduli with the composition is displayed in Figures 3e and f. We can use this composition variation for a better understanding of the actual contribution of the hybrid structure to the mechanical properties. For the 1D hybrid lattice, different composition dependencies were proposed for the in-plane and cross-plane effective moduli.^52^ For the in-plane moduli, it is a linear interpolation of the volume-weighted elastic properties of the individual layers, as is also implied by the experimental *E*_∥_ in Figure 3e. *E*_∥_(ϕ_Hec_) is well represented by a linear regression using *E*_∥,Hec_ = 179 GPa and *E*_PEG_ = 6.1 GPa, suggesting a weak impact of the predominantly parallel Hec/PEG interfaces; *E*_PEG_ = 6.1 GPa is a meaningful value for glassy or crystalline polymers in a stretched conformation. The large elasticity mismatch between the Hec and soft polymer layers obscures any conclusion on the polymer specificity (PVP vs PEG) on *E*_∥_. Furthermore, the conformity of *E*_∥_ to the predictive mixing rule suggests that *E*_∥_ is an inherent material property that should monotonically depend on the filling ratio.

Along the 1D periodicity direction, the phonon dispersion relationship at long wavelength predicts Wood’s law for the composition dependence of the cross-plane modulus *E*_⊥_.^52,53^ However, as previously reported for Hec/PVP Bragg stacks,^32^Figure 3e implies failure of this law for *E*_⊥_ because the predicted dependence is exhibits a concave form (Figure S9) in contrast to the experimental convex form. For the high filling fractions (>50%), we find a plateau region with *E*_⊥_ comparable to that at 100% Hec. The very high filler contents applied here cause a severe confinement of the thin polymer layers. As a consequence, the polymer interlayers fail to efficiently decouple adjacent nanosheets in the stack, which would be required for energy dissipation.

Down to a content of ∼50 vol % Hec, *E*_⊥_ is unaffected by the presence of a confined polymer, whereas at <50 vol % Hec, the increasing volume fraction of PVP does lead to a decreasing *E*_⊥_ but stronger than the Wood’s law prediction (Figure S9). The clearly different composition dependence of *E*_⊥_ and *E*_∥_ emphasizes the role of the interface density solely for the cross-plane elasticity (above ∼50 vol % Hec). Because of this difference, the elasticity anisotropy, *E*_∥_/*E*_⊥_, increases with the Hec content from 4.6 at 54 vol % Hec to 6.7 at 82 vol % Hec (Table 1). This extraordinary anisotropy is induced by the structure and depends on the large elastic mismatch of the components. Moreover, it is rather polymer-unspecific, which becomes obvious when the PVP and PEG samples are compared.

Analogous to *E*_∥_, the in-plane torsional shear modulus, *G*_12_, lies between the values of the two bulk components and displays an effective medium behavior (Figure 3e,f). Consistently, the in-plane moduli show no difference for the DS and Hec samples. Both moduli (i.e., *E*_∥_ and *G*_12_) increase by about 50% as the Hec content increases from 54 to 82 vol %. The bulk modulus *K* increases only by about 15% and is comparable to *E*_⊥_ with a similar saturation above ∼60 vol % Hec because both are primarily controlled by the elastic constant *C*_33_. Consistently, the cross-plane moduli, *E*_⊥_ and *G*_13_, are hardly influenced by the composition (Figure 3e,f). The sliding shear modulus, *G*_13_, remains low but is higher (almost by a factor of 2) for Hec/PEG than Hec/PVP hybrid Bragg stacks. This is the only indication of a polymer specificity that we see in the two investigated systems. We attribute this to the more compact PEG intercalation between the Hec sheets with less free volume.

The Poisson’s ratio (Table 1) ν_12_ represents how the sample shrinks in the “2” direction due to an applied stretching strain in the “1” direction. Note that the “1–2” plane means the sample plane, and hence the high ν_12_ value is polymer-like. The second Poisson’s ratio ν_31_ expresses how the sample expands in the “1” direction due to an applied compressing strain in the “3” direction. Hence, the almost zero value of ν_31_ implies negligible deformation of the PEG layer along the “1” direction; we note that wine corks have a Poisson’s ratio of nearly zero. Among the different elasticity parameters, *G*_13_ and ν_12_ reflect the properties of the confined polymer.

### In-Plane and Cross-Plane Thermal Conductivities

**Figure 4:**
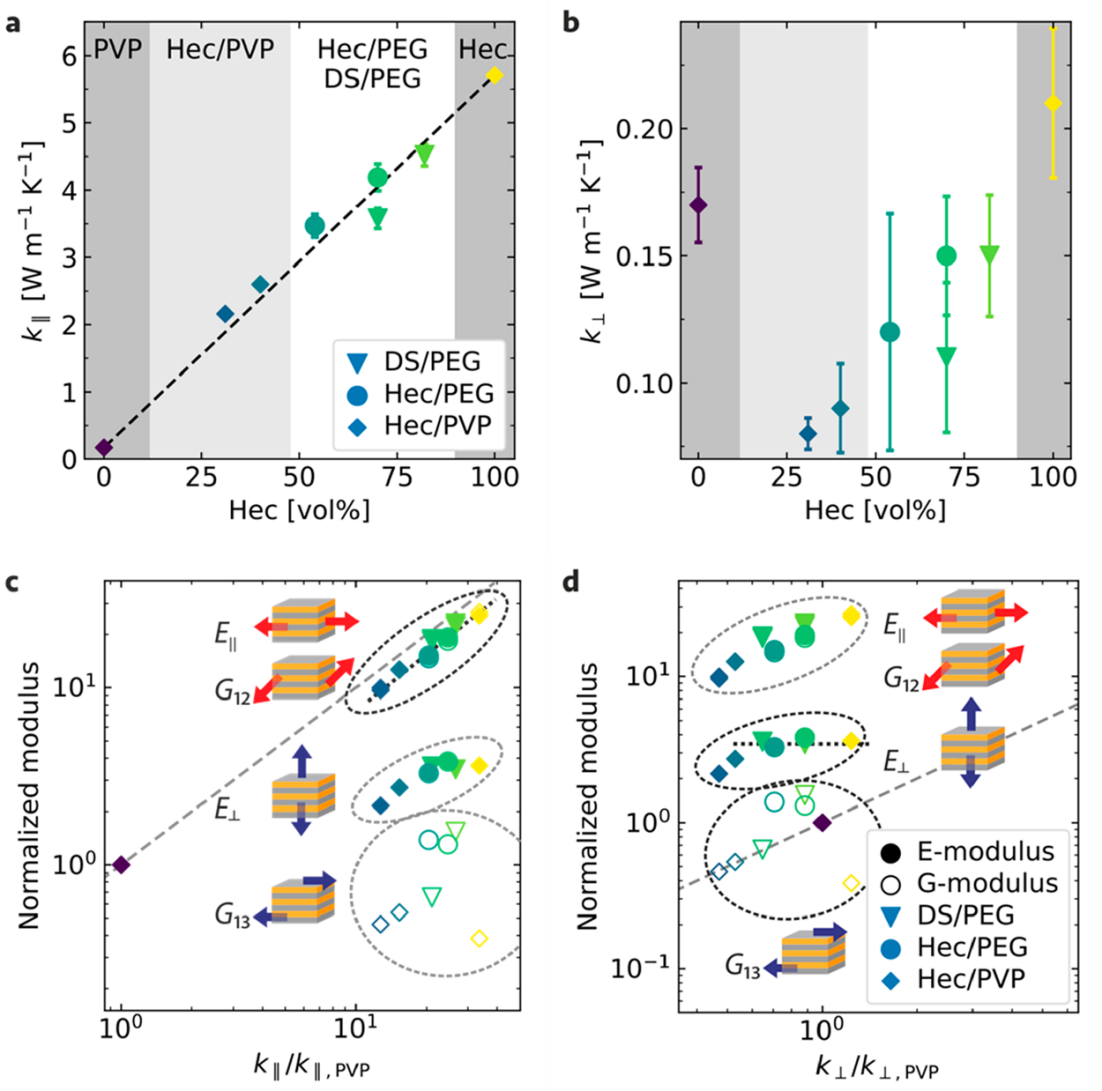
Anisotropic thermal transport and correlation to elasticity. Thermal conductivity of crystalline hybrid Bragg stacks in the (a) in-plane and (b) cross-plane directions. Analogous Hec/PVP samples are shown as diamonds.^32^ Samples consisting of Hec/PEG and DS/PEG are represented by circles and triangles, respectively. Correlation between the relative changes in the mechanical moduli and the corresponding relative changes in the (c) in-plane and (d) cross-plane thermal conductivities. All data are normalized by the respective value of the pure PVP polymer. The symbol colors indicate the volume fraction of Hec.

Another approach to characterize the anisotropy of the hybrid Bragg stacks is to measure their thermal transport properties. Therefore, we characterized the in-plane and cross-plane thermal conductivities using lock-in thermography and photoacoustic measurements, respectively.^54−56^ Analogous to the BLS characterization, we include the previously reported Hec/PVP system with one (40 vol % Hec) and two (31 vol %) layers of PVP, respectively. The in-plane thermal conductivity κ_∥_ follows a linear composition dependency. According to the parallel thermal resistance network model, κ_∥_ = κ_PEG_ + (κ_Hec(DS)_ – κ_PEG_)ϕ_Hec_, with κ_Hec(DS)_ and κ_PEG_ being the thermal conductivities of Hec (or DS) and PEG, respectively, and ϕ_Hec_ the Hec volume fraction (dashed line in Figure 4a). This behavior confirms the reported behavior of Hec/PVP hybrid Bragg stacks^32^ and the in-plane elastic moduli (Figure 3e,f). Apparently, neither the delamination and reassembly process of Hec/DS nor the extreme polymer confinement influences the in-plane transport of the two components.

Because of the large thermal conductivity contrast between the polymers and Hec/DS, we do not see a polymer-specific behavior. The cross-plane thermal conductivity κ_⊥_, however, decreases gradually from 0.21 to 0.08 W m^–1^ K^–1^ as the Hec composition decreases from 100 to 31 vol % (Figure 4b), which contrasts with the plateau of κ_⊥_ (∼0.08 W m^–1^ K^–1^) in the Hec/PVP system.^32^ According to the serial thermal resistance network model, 1/κ_⊥_ = (−1/κ_PEG_ + 1/κ_Hec_ + 2*R*_Hec/PEG_/*d*_⊥,Hec_)ϕ_Hec_ + (1/κ_PEG_), with *R*_Hec/PEG_ and *d*_⊥,Hec_ being the thermal resistance per unit area of a Hec/PEG interface and the thickness of one Hec layer, respectively. The increasing κ_⊥_ with ϕ_Hec_ then suggests that −1/κ_PEG_ + 1/κ_Hec_ + 2*R*_Hec/PEG_/*d*_⊥,Hec_ < 0. Therefore, the cross-plane thermal resistances of the Hec/PEG samples are not dominated by the Hec/PEG interfaces, unlike the situation of the Hec/PVP samples, where the stronger influence of the interfaces leads to a plateau of κ_⊥_ in the considered Hec volume fraction range. The different effects of the Hec/PEG and Hec/PVP interfaces could stem from the different physical states of PEG and PVP because of the vastly different glass transition temperatures (210 vs 445 K) and the stronger complexation between PEG and Na^+^.^42^ Both effects can influence the chain mobility within the confined space and, consequently, lead to a different interfacial adhesion between the Hec/PEG and Hec/PVP interfaces, respectively. In the case of PEG, the decrease can also be driven by the introduction of different types of interfaces in the hybrid material. While the samples with 1PEG involve only polymer–hectorite interfaces, for 2PEG an additional polymer–polymer interface is introduced (if a stretched PEG conformation is assumed). One should further note that κ_⊥_ is more strongly reduced for DS than for Hec nanosheets. We attribute this to an additional type of interface characteristic for the DS filler, namely, the NH_4_^+^ cation interlayer “gluing” two Hec sheets to form a DS.

In Figure 4c,d, we provide correlation plots between the thermal transport and mechanical properties. We should note, however, that the thermal conductivity and elastic moduli are not generally directly correlated for all materials because of their different influencing factors, but many materials of a high thermal conductivity are also accompanied by high elastic moduli. Therefore, it is worth studying the correlation between the thermal conductivity and elastic moduli of the Hec/PEG Bragg stacks. For the in-plane direction, we find a direct correlation between κ_∥_, *E*_∥_, and *G*_12_, which is emphasized by the black dashed line (Figure 4c). We used the thermal conductivity and mechanical moduli of PVP as a reference for the correlation plots of both systems. This is justified because the bulk PVP microstructure is amorphous and, therefore, more similar to the confined case, as we would expect for the semicrystalline bulk PEG. This direct correlation demonstrates that the in-plane mechanical moduli and in-plane thermal transport are interrelated with each other.

Even more intriguing is the comparison of the composition sensitivity between the mechanical and thermal properties in the cross-plane direction. Whereas *E*_⊥_ remains almost constant for the highly filled Hec/PEG and DS/PEG samples (>50 vol %), a monotonic decrease with decreasing filler content can be observed in the case of κ_⊥_. This decrease is even more dramatic considering that κ_⊥_ of the hybrid films is consistently lower than that of the pure components. We can, therefore, conclude that the interface chemistry has a significant contribution to the cross-plane heat transport. As outlined above, the cross-plane thermal transport continuously decreases with the introduction of additional types of interfaces (Hec–PEG, PEG–PEG, and Hec–Hec). The cross-plane mechanical moduli (*E*_⊥_ and *G*_13_) are not influenced by these different types of interfaces. This also becomes apparent in the correlation plots (Figure 4d), where *E*_⊥_ versus κ_⊥_ exhibits a flat line (black dashed line) for filler contents of >50 vol %. This difference in sensitivity to the presence of the nanostructure and interfaces is further translated into a 4–5-fold increased anisotropy compared to the mechanical anisotropy. The similar composition dependencies of κ_⊥_ and κ_∥_ result in a roughly constant anisotropy ratio (κ_∥_/κ_⊥_ = 28–33; Table 2) of the hybrid Bragg stack films.

**Table 2:** Thermal Measurement Results of the Hybrid Bragg Stack Films

sample ID	Hec (vol %)	ρ (g cm^–3^)	*c*_p_ (J g^–1^ K^–1^)	α_∥_ (mm^2^ s^–1^)	κ_∥_ (W m^–1^ K^–1^)	κ_⊥_ (W m^–1^ K^–1^)	κ_∥_/κ_⊥_
Hec/2PEG	54	2.01 ± 0.03	1.27 ± 0.06	1.358 ± 0.021	3.47 ± 0.17	0.12 ± 0.05	29
Hec/1PEG	70	2.29 ± 0.03	1.12 ± 0.05	1.636 ± 0.031	4.19 ± 0.20	0.15 ± 0.03	28
DS/2PEG	70	2.01 ± 0.06	1.08 ± 0.03	1.645 ± 0.020	3.58 ± 0.15	0.11 ± 0.03	33
DS/1PEG	82	2.35 ± 0.05	1.04 ± 0.03	1.854 ± 0.019	4.52 ± 0.16	0.15 ± 0.03	30

### Mechanical Testing

**Figure 5:**
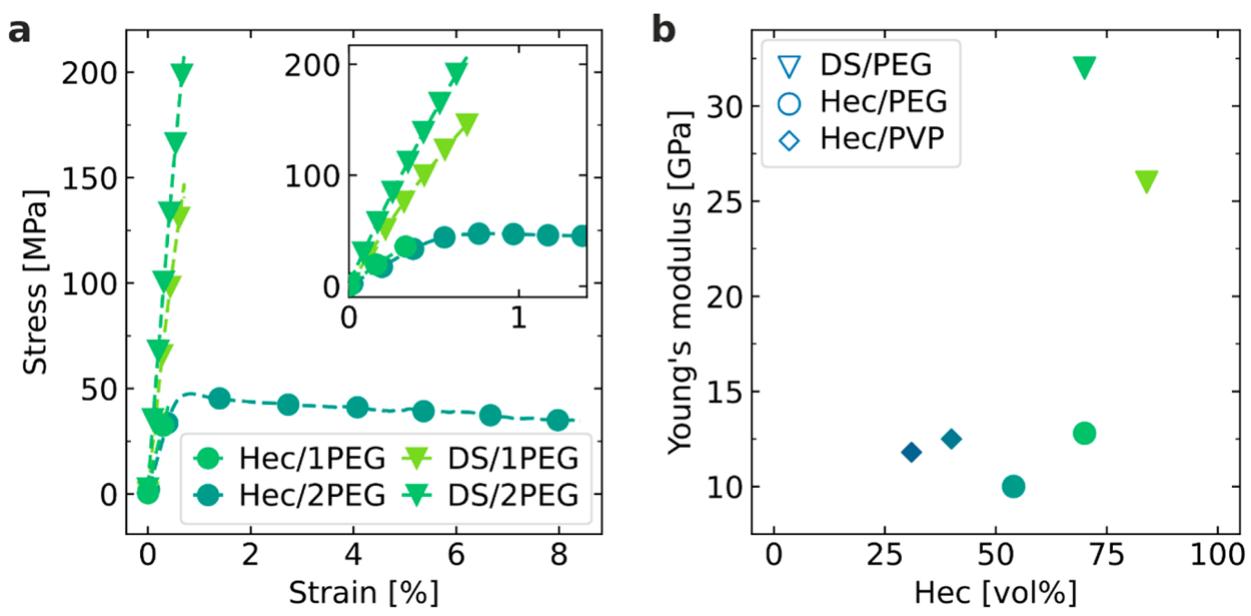
Bulk mechanical properties by tensile testing. (a) Stress–strain curves of the hybrid Bragg stack Hec/PEG films indicated in the plot. Influence of the thickness of the filler (Hec vs DS) and of the polymer layer (1PEG vs 2PEG). Inset: Zoom-in of the data in the main plot in the low strain range. (b) Young’s modulus as a function of the Hec filler content for PEG and PVP hybrids.^32^

The macroscopic mechanical properties are obtained from the stress–strain curves of the hybrid Bragg stack films measured at 53% relative humidity, as shown in Figure 5a. First, Young’s modulus *E* (10–30 GPa in Figure 5b) and strength σ (40–200 MPa; Figure 5a and Table S2) are interestingly high because comparable values in nanocomposites are only achieved with high *T*_g_ polymers^16,57^ in combination with cross-linking^10,58^ or by combining low *T*_g_ polymers with hydrogen-bonding networks.^24,59^ Second, the correlation between *E* and ε (%) at different Hec or DS contents is not straightforward. In contrast to the other Hec/PEG hybrid materials, Hec/2PEG displays a plastic deformation at σ = 48 MPa and breaks at a high elongation of 8.4% (inset of Figure 5a). This transparent nacre mimetic Hec/2PEG can be folded like paper (Figure S5) without breaking. Hec/2PEG features the smallest *E* value but is still comparable to the Hec/1PEG system, which has the smallest elongation at break (ε = 0.4%). Hec/2PEG, consequently, represents a unique and singular property that is elusive to a predictive material engineering based on the effective material properties. This finding suggests that macroscopic stress–strain measurements alone are inappropriate to guide the structure property optimization of nacre mimetics. Third, *E* does not follow the expected increasing trend with the filler content. While DS hybrids are indeed stronger than the corresponding single Hec counterparts, DS/1PEG with 82 vol % Hec is weaker than DS/2PEG (70 vol % Hec; Table S2). The missing correlation of *E* with the filler content suggests that even in the linear-elastic region at very low strain, nonlocal contributions, for instance, by defects, are significant.

Such defects do not play a role in BLS measurements, unless they feature a length scale of a few 100 nm, which we can rule out based on the structural integrity of our hybrid materials. It is still surprising that the in-plane Young’s modulus, *E*_∥_, from BLS (Figure 3e) is about an order of magnitude higher than the value obtained from the tensile test experiment (Figure 5b). Both techniques should, in principle, yield the same *E*_∥_ for glassy, frequency-independent systems. The observed large disparity demonstrates the inapplicability of the stress–strain experiments to accessing the inherent “true” material mechanics. In addition, the weak composition dependence and the large increase of *E* for the samples with DS fillers in Figure 5b corroborate the notion that the macroscopic *E* cannot be determined from *E*_∥_ from the microscopic BLS probe. Although *E* is obtained from unidirectional stretching, it is remarkable that its value is of the same order of magnitude as the cross-plane *E*_⊥_ from the BLS experiment and that both also exhibit a similar weak composition dependence (Figures 5b and 3e). In the case of BLS, we do find—depending on the direction—a systematic variation of the elastic moduli.

The macroscopic mechanical properties based on stress–strain measurements show considerably lower values, thus emphasizing the role of flaw tolerance. Still, the results indicate a high sensitivity to the exact nanoscale structure; when two PEG layers are kept in the interlayer spaces, a brittle-to-ductile change is observed by replacing DS fillers with Hec fillers. These results demonstrate that the complexity hampers a rational design of the multidimensional property space of hybrid Bragg stacks. Further research is needed to bridge the gap between the inherent “true” hybrid properties on the submicrometer scale and those in the macroscopic world.

## Conclusion

This work highlights the rich material parameters and anisotropy property space of seemingly simple 1D hybrid materials consisting of alternating inorganic clay nanosheets and polymer layers. We construct nanoscopically well-defined periodic and anisotropic layered materials and use advanced characterization techniques to assess the ultimate properties of the stacks. We use fluorohectorite clay that uniquely allows osmotic swelling, which is central to well-defined structures allowing discrete “quantized” compositions of one or two clay layers to be combined with one or two layers of PEG polymer. This clay allows, for the first time, comparatively small volume fractions of polymers within the nanosheets to form uniform single-phase 1D hybrid Bragg stacks. The anisotropic elastic moduli are characterized using BLS. Notably, the composition consisting of 82 vol % DS fillers and 18 vol % 1PEG layers shows a Young’s modulus of 161.8 GPa, which suggests almost ideal stress transfer parallel to the clay nanosheets. The corresponding elastic modulus in the transverse direction is 6.7 times smaller, indicating strong mechanical anisotropy. The anisotropy is also manifested in the thermal conductivity, where it is 4.52 W m^–1^ K^–1^ in the direction of the platelets and 30 times less in the transverse direction. The macroscopic mechanical properties based on stress–strain measurements do not reach such high Young’s modulus values, possibly because of the presence of defects. High sensitivity to the exact nanoscale structure is also emphasized by comparing the two samples with 2PEG layers in the interlayer spaces, where a brittle-to-ductile change is observed by replacing the double-layer clays with single-layer clays.

The results pave the way to unravelling the complexity of designing hybrid stacks. Maximizing the filler content and the interaction of the polymer chains with the ceramic surface tends to result in brittle materials with little flaw tolerance. The challenge of translating the superior nanoscale mechanical properties to the macroscopic behavior seems to be a hierarchical integration of such Bragg stack into optimized matrixes with tailored gradient-type interfaces between them. This may eventually lead to combined molecular and nanostructured design approaches that allow for an independent adjustment of the mechanical and thermal properties and may even be expanded to realize additional functionalities, such as magnetism, molecular, or electric transport.

## Experimental Section/Methods

### Materials

The synthetic clay [Na_0.5_]^inter^[Mg_2.5_Li_0.5_]^oct^[Si_4_]^tet^O_10_F_2_) was obtained via melt synthesis followed by long-term annealing, according to an established procedure.^38,41^ The synthesis of ordered heterostructures (interstratifications) consisting of strictly alternating sodium and ammonium interlayers was performed according to an already published procedure.^29^ PEG (*M*_w_ = 1500 g mol^–1^) was provided by Sigma-Aldrich.

### Film Preparation and Characterization

The as-synthesized fluorohectorites and heterostructures were delaminated by immersing them in Millipore water (0.4 vol %), producing nematic liquid-crystalline suspensions of Hec (1 nm) and DS (2 nm), respectively. For a reduction of the original diameters of Hec and DS, the suspensions were sonicated with an ultrasonic horn. PEG was dissolved in Millipore water (0.8 vol %) and added in the desired weight ratio. The suspension was mixed for at least 1 day in an overhead shaker. The quality of the suspension, in terms of homogeneity, was crosschecked by SAXS. Self-supporting films were prepared by employing a fully automatic spray-coating system. Every spraying cycle was followed by a drying cycle of 90 s at a temperature of 40 °C. The films were characterized by XRD, TEM, and thermogravimetric analysis. The mechanical characteristics were determined by tensile testing with a ZwickRoell testing machine equipped with a 20 N load cell. In contrast to the pure components PEG and Hec, nanocomposites with such high filler contents have been shown to be barely sensitive to ambient relative humidities.^21^ Consequently, we conducted all of our experiments at ambient humidity conditions, without further environmental control. Additional information about the sample preparation and characterization methods can be found in sections S1 and S2.

### BLS

BLS records the phonon dispersion, ω(**q**), by detecting the Doppler frequency shift, ω, of the inelastically scattered light by sound waves (“phonons”) with a wave vector, **q**. We recorded BLS spectra by utilizing three scattering geometries (transmission, reflection, and backscattering) and two polarization configurations of the incident (λ = 532 nm) and scattered (polarized, VV; depolarized, VH) light, which allowed us to establish the nature of the observed phonons. We varied the incidence angle to obtain the direction-dependent sound velocities necessary for determination of the anisotropic elasticity. The elastic stiffness tensor was obtained from the representation of the direction-dependent sound velocities by the Christoffel equation assuming transverse isotropy. The characteristic Young’s moduli, shear moduli, and Poisson’s ratios of the Bragg stacks were subsequently calculated. More details can be found in sections S3 and S4.

### In-Plane Thermal Conductivity Measurement Materials

Lock-in thermography measures the temperature spreading across the free-standing samples upon thermal excitation by a line-laser beam with a modulated intensity. To prevent convective heat losses, the experiments were conducted in a vacuum chamber. The amplitude and phase data were extracted from the temperature distribution perpendicular to the laser line. The thermal diffusivity was then fitted by the slope method for thermally thin films.^55^ With the density, determined by helium pycnometry, and the specific heat, determined by differential scanning calorimetry, the thermal conductivity was calculated. More details are provided in section S5.

### Cross-Plane Thermal Conductivity Measurements

The photoacoustic method uses a modulated laser beam to heat the sample periodically. The surface temperature is converted in an acoustic wave propagating into a gastight cell above the sample, filled with helium at 20 psi. A sensitive microphone detected the phase shift between the acoustic signal and modulated heat source by a lock-in amplifier. The frequency-dependent phase shift was then compared to a multilayer model (gold transducer, sample, and quartz substrate), assuming 1D heat transfer. With the film thickness determined by laser scanning microscopy, the thermal conductivity was obtained, neglecting thermal contact resistances. More details are provided in section S5.

## Supporting Information Available

Sample preparation, characterization of the suspensions and films, BLS, transversely isotropic elasticity model, and thermal measurements (PDF)
