# Supplementary material for: Nanoscale-Structured Hybrid Bragg Stacks with Orientation- and Composition-Dependent Mechanical and Thermal Transport Properties: Implications for Nacre Mimetics and Heat Management Applications
Source: ACS Appl Nano Mater. 2022 Mar 2;5(3):4119–29. doi: 10.1021/acsanm.2c00061 (PMC8961742; doi:10.1021/acsanm.2c00061)
Supplement: Supplementary file 1 — an2c00061_si_001.pdf [file an2c00061_si_001.pdf]

Supporting Information

Nanoscale-Structured Hybrid Bragg Stacks with Orientation- and Composition-Dependent Mechanical and Thermal Transport Properties: Implications for Nacre Mimetics and Heat Management Applications

Theresa Dörres[§,†], *Malgorzata Bartkiewicz*[§,‡], *Kai Herrmann*[§,†], *Marius Schöttle*[†], *Daniel Wagner*[†], *Zuyuan Wang*[#], *Olli Ikkala*[¶], *Markus Retsch*[†,*], *George Fytas*[‡,*], *Josef Breu*[†,*]

† Bavarian Polymer Institute and Department of Chemistry, University of Bayreuth, Universitätsstr. 30, 95440 Bayreuth, Germany.

‡ Max Planck Institute for Polymer Research, Ackermannweg 10, 55128 Mainz, Germany.

# School of Mechanical and Electrical Engineering, University of Electronic Science and Technology of China, Chengdu, Sichuan 611731, China.

¶ Department of Applied Physics, Aalto University, P.O. Box 15100, FI-00076, Espoo, Finland.

Corresponding Authors

Josef Breu: josef.breu@uni-bayreuth.de.

George Fytas: fytas@mpip-mainz.mpg.de.

Markus Retsch: markus.retsch@uni-bayreuth.de.

S1. Sample preparation

Materials

The synthetic clay sodium fluorohectorite (Hec, $[Na_{0.5}]^{inter}[Mg_{2.5}Li_{0.5}]^{oct}[Si_4]^{tet}O_{10}F_2$) was synthesized by melt synthesis followed by long-term annealing, according to an already published procedure.[1, 2] The material featured a cation exchange capacity of 1.18 mmol g^{-1}.[1, 2] Upon immersion in deionized water, the pristine material swells osmotically producing a nematic suspension with nanoplatelets of mean diameters of ≈ 18 µm being separated by > 100 nm at 0.4 vol%.[3]

The synthesis of double stacks (DS), consisting of strictly alternating sodium and ammonium interlayers was performed according to an already published procedure.[4] To reduce the original diameter into the range of natural montmorillonites, a 0.4 vol% suspension of delaminated Hec or DS was sonicated for 15 minutes in an ice bath applying a UIP 1000hd (Hielscher Ultrasonic GmbH, Germany) equipped with a ultrasonic horn BS2d22 and a booster B2-1.2, at 20 kHz with a maximal output power of 1000 W.

The sonicated suspensions were diluted (0.0004 vol%), drop cast on a plasma-treated silicon wafer and sputtered with 10 nm carbon for scanning electron microscopy (SEM) applying a Zeiss Ultra plus (Carl Zeiss AG, Germany) at an operating voltage of 3 kV. *ImageJ* was used to evaluate the average diameter of 150 nanoplatelets. (Figure S1). The average diameters of Hec and DS are 340 and 406 nm, respectively.

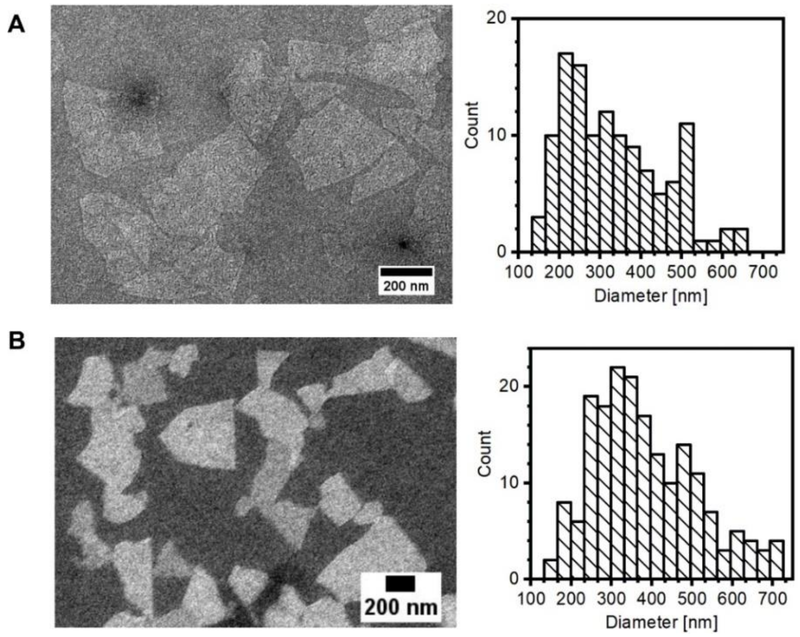

Figure S1. Typical SEM images and histograms of the platelet diameter distribution of sonicated (A) Hec and (B) DS as evaluated by *ImageJ*.

Film preparation

An aqueous Polyethylene glycol (PEG-1500, M_w = 1500 g mol^{-1}, Sigma Aldrich) solution (0.8 vol%) was added in appropriate amounts to obtain the desired volume ratio of Hec or DS to PEG. To assure homogenization, the suspension was mixed for 1 day in the overhead shaker. The self-supporting films were prepared by spray coating. The fully automatic spray coating system was equipped with a SATA 4000 LAB HVLP 1.0 mm spray gun (SATA GmbH & Co. KG, Germany). Suspensions were sprayed on a corona-treated polyethylene terephthalate (PET) foil (Optimont 501, Bleher Folientechnik, Germany). The spraying and nozzle pressures were set at 2 and 4 bar, respectively. The round per flat fan control was set to 6 with a flow speed of 3 mL s^{-1}. The distance between the spraying gun and the substrate was 17 cm. The thickness of the suspension layer applied in one spraying step is about 2 µm which corresponds to about 20 nm dry film thickness. For drying the suspension layer, the sample was stopped under infrared lamps until evaporation of the solvent was complete. After every spraying cycle, a drying cycle of 90 s with a temperature of 40 °C took place. The spraying/drying cycle was repeated until the desired barrier film thickness of 30 µm was obtained. Afterward, the film was dried at 60 °C for 7 days, and peeled off from the PET foil for achieving self-supporting films. For characterization by photoacoustic analysis, thinner films on the order of a few µm were spray coated onto clean 1 mm thick quartz slides.

In total, we prepared four different samples: Hec/2PEG, Hec/1PEG, DS/2PEG, DS/1PEG. The nomenclature is based on the two different volume contents of PEG. A detailed explanation is given in Table S1.

S2. Characterization of the suspensions and films

Small Angle X-Ray Scattering (SAXS)

SAXS data were measured using the small-angle X-ray system "Double Ganesha AIR" (SAXSLAB, Denmark). The X-ray source of this laboratory-based system is a rotating anode (copper, MicroMax 007HF, Rigaku Corporation, Japan), providing a micro-focused beam. The data were recorded by a position-sensitive detector (PILATUS 300 K, Dectris). To cover the range of scattering vectors different detector positions were used. The measurements of the suspensions were done in 1 mm glass capillaries (Hilgenberg, code 4007610, Germany) at room temperature. To improve the detection limit of the in-house machine, the suspensions were first concentrated by centrifugation at 10,000 rpm for 1 hour. The data were radially averaged and background corrected. As background, a water-filled capillary was used.

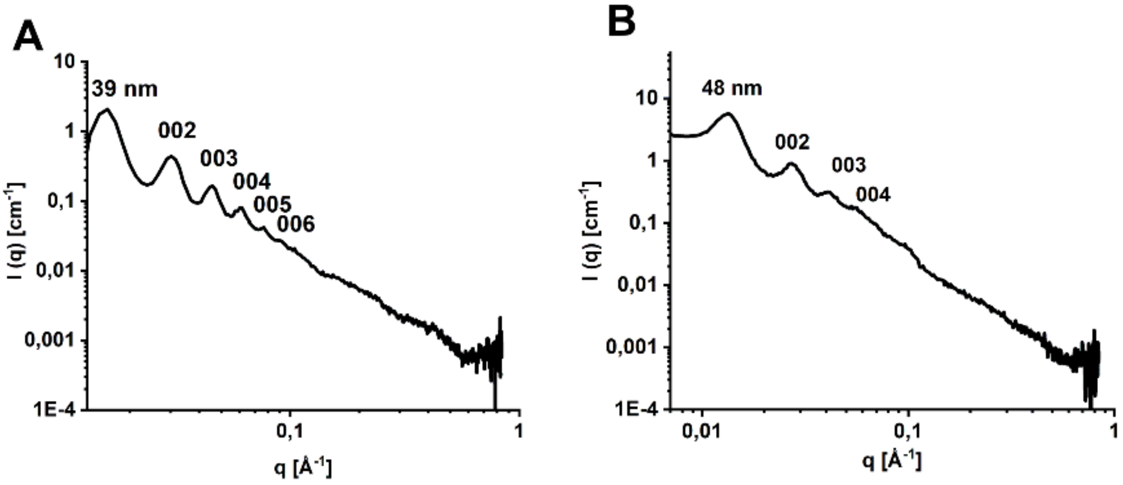

Figure S2. One-dimensional SAXS pattern of the concentrated gel samples A) Hec/2PEG, B) DS/2PEG. The varying layer separations observed are related to the clay content of the suspensions rather than the Hec/PEG ratio.

X-Ray diffraction (XRD) analysis

XRD patterns for the films were recorded in Bragg-Brentano-geometry on an Empyrean diffractometer (PANalytical B.V.; the Netherlands) using Cu K_α radiation (λ = 1.54187 Å). The self-supporting films were placed on glass slides (Menzel-Gläser; Thermo Scientific). Before the measurements, samples were dried at 100 °C for one week in a vacuum chamber.

As a measure of the quality of the one-dimensional crystallinity of the films, the coefficient of variation (CV) and the full width at half maximum (FWHM) were determined (Table S1). Large CV-values (> 3%[4]) and large FWHM indicate non-rationality of the diffraction pattern as

caused by a random interstratification of different interlayer heights, which represent defects in the periodicity.

Table S1. Overview of the structural and chemical characterization

sample	nominal Hec:PEG ratio (wt:wt)	nominal Hec:PEG ratio (vol:vol)	PEG content * (wt:wt)	PEG content ** (vol:vol)	Nominal d-spacing (nm)	Observed d-spacing (nm)	CV (%)	FWHM (°2θ)
Hec/2PEG	73:27	54:46	27	46	1.77	1.77	0.3	0.3-0.5
Hec/1PEG	84:16	70:30	16	30	1.38	1.38	0.6	0.5-0.9
DS/2PEG	84:16	70:30	16	30	2.77	2.79	1.6	0.4-0.8
DS/1PEG	91:9	82:18	9	18	2.38	2.39	1.0	0.5-1.0

* determined by TGA (Figure S4); ** recalculated from vol% PVP assuming bulk density

The FWHM and CV criteria were applied to examine the stoichiometry of the hybrid films. Even slight deviations from the ideal compositions can lead to significant increase in both CV and FWHM of the *001* reflection (Figure S3).

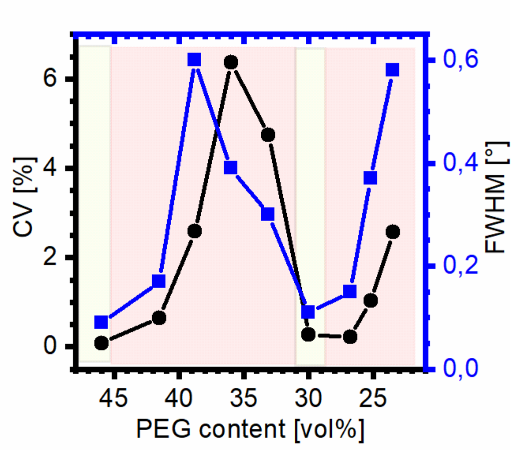

Figure S3. Optimization of the PEG content of Hec/2PEG and Hec/1PEG to improve the 1D crystallinity of hybrid Bragg stacks. The highest 1D crystallinity corresponds to minima in CV and FWHM, which are observed at 46 and 30 vol% for Hec/2PEG and Hec/1PEG, respectively.

Thermogravimetric analysis (TGA)

The Hec to PEG ratios were cross-checked (Table S1) for the dried films by thermogravimetric analysis (TGA), using a Mettler Toledo SDTA851 equipped with the gas control unit

TS0800GC1 (Mettler Toledo, USA). Changes in mass observed upon heating in synthetic air up to 900 °C were attributed to the combustion of PEG. (Figure S4).

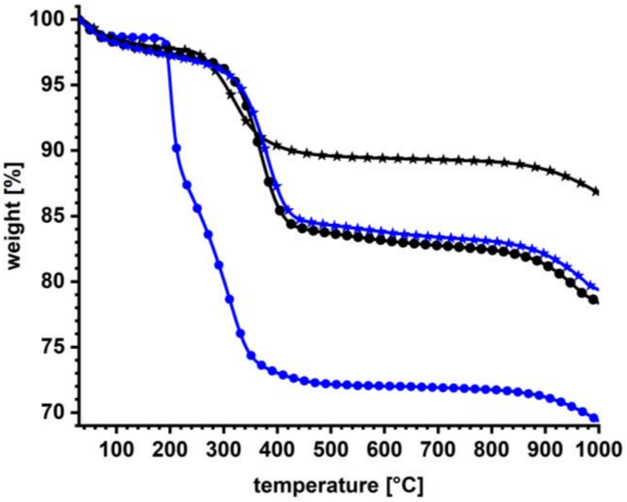

Figure S4. TGA curves of Hec/2PEG (blue curve, circle), Hec/1PEG (blue curve, asterisk), DS/2PEG (black curve, circle) and DS/1PEG (black curve, asterisk). The weight loss below 150 °C corresponds to adsorbed water.

Transmission electron microscopy (TEM)

TEM images of the self-supporting films were taken on a JEOL JEM-2200FS (JEOL GmbH, Germany) at an acceleration voltage of 200 kV. Cross-section pictures of the self-supporting films were prepared with a Cryo Ion Slicer IB-09060CIS (JEOL, Germany).

Tensile testing

The stress-strain curves were recorded with a ZwickRoell material testing machine of the type BT1-FR0.5TN.D14 (ZwickRoell, Ulm, Germany) equipped with a 20 N load cell (Xforce HP, ZwickRoell, Ulm, Germany). The test strips had a dimension of 20 mm x 2 mm x 30 µm. The test speed was set to 0.5 mm/min. Before testing, the test strips were conditioned in a desiccator with a relative humidity of 53% RH for 7 days. A minimum of 7 test strips was measured for every sample.

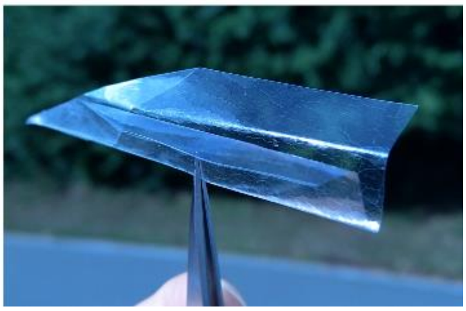

Figure S5. Transparent and flexible self-supporting films, some of them allowing folding without breaking (Hec/2PEG).

Table S2. Bulk mechanical properties obtained by tensile testing at 53% RH. The mean and standard deviation values are calculated based on seven individual measurements.

sample	Hec:PEG ratio, (vol:vol)	Young's modulus, *E* (GPa)	Maximum tensile strength, σ (MPa)	Elongation at break, ε (%)	Work of fracture (MJ/m^3)
Hec/2PEG	54:46	10 ± 1	48 ± 5	8.4 ± 2.0	3.4 ± 0.9
Hec/1PEG	70:30	13 ± 1	42 ± 8	0.4 ± 0.1	0.1 ± 0.0
DS/2PEG	70:30	32 ± 3	202 ± 16	0.7 ± 0.1	0.8 ± 0.1
DS/1PEG	82:18	26 ± 3	149 ± 19	0.7 ± 0.1	0.6 ± 0.1

S3. Brillouin light spectroscopy (BLS)

BLS is an optical, nondestructive, and noninvasive technique which measures the inelastic scattered light caused by thermally excited hypersonic (GHz) phonons. The scattering wave vector $\mathbf{q}$ is defined as $\mathbf{q} = \mathbf{k}_s - \mathbf{k}_i$, where $\mathbf{k}_i$ and $\mathbf{k}_s$ are the wave vectors of the incident and scattered light, respectively. The polarization of the incident and scattered light was selected to be either vertical (V) or horizontal (H) to the scattering plane defined by $\mathbf{k}_i$ and $\mathbf{k}_s$. To take full advantage of the vector nature of the phonon wave vector we used three scattering geometries, transmission, reflection, and backscattering. They allowed us to probe phonon propagation in directions parallel, normal, and oblique to the sample film, respectively. For the transmission and reflection geometries the laser source was mounted on a goniometer and rotated around the sample. In the transmission geometry, $\mathbf{q}$ lies in the plane of the sample film with a

magnitude, $q_{||} = \frac{4\pi}{\lambda}\sin\beta$, where β is the incident angle and λ (= 532 nm) the wavelength of the laser beam in vacuum. In the reflection geometry, $\mathbf{q}$ is directed normal to the sample film with a magnitude, $q_{\perp} = \frac{4\pi}{\lambda}\sqrt{n^2 - \sin^2\beta}$, where n is the sample's refractive index. In the backscattering geometry, the incident and scattered light follow the same path, and $q_{\mathrm{bs}} = \frac{4\pi}{\lambda}n$. The BLS spectra (Figure S6) were recorded by a six-pass tandem Fabry-Perot interferometer and the frequency shift (*f*) of the phonons at the three different geometries was obtained from the Lorentzian representation of the BLS data. The longitudinal c_{L} (transverse c_{T}) phonon sound velocity is computed from the dispersion $f(q)$ (Figure S7) relation obtained from the VV (VH) BLS spectra. For linear acoustic dispersion (in the absence of relaxation phenomena), $c = 2\pi f / q$, and moreover for an elastically isotropic sample, the longitudinal (transverse) sound velocity c_{L} (c_{T}) is independent of the propagation direction. In this case, the in-plane (transmission geometry) and out-of-plane (reflection geometry) $f(q)$ coincide, leading to the determination of the refractive index, n. In the case of linear acoustic dispersion but elastically anisotropic samples, both sound velocities are direction-dependent.

S4. Transversely isotropic elasticity model

It is well known that the elastic tensor for an anisotropic material contains 21 independent components. We assume transversely anisotropic films with the axis of symmetry normal to the film which reduces the number of independent tensor components to 5 (e.g., C_{11}, C_{13}, C_{33}, C_{44}, C_{66}). The elastic stiffness tensor has the following form in the Voigt notation[6]

$$\mathbf{C} = \begin{pmatrix} C_{11} & C_{12} & C_{13} & 0 & 0 & 0 \\ C_{12} & C_{11} & C_{13} & 0 & 0 & 0 \\ C_{13} & C_{13} & C_{33} & 0 & 0 & 0 \\ 0 & 0 & 0 & C_{44} & 0 & 0 \\ 0 & 0 & 0 & 0 & C_{44} & 0 \\ 0 & 0 & 0 & 0 & 0 & C_{66} \end{pmatrix}, \quad \text{(S1)}$$

where $C_{66} = (C_{11} - C_{12})/2$.

Considering the symmetry, we take into account only $\mathbf{q}$ vectors in one plane containing the axis of symmetry, which allows us to define the direction as $0° \le \alpha \le 90°$ - the angle between $\mathbf{q}$ and the normal to the sample film. For a particular direction, there exist one quasi-longitudinal (Q-L) mode, one quasi-transverse (Q-T) mode measured in the VV polarization configuration, and one pure-transverse (P-T) mode measured in the VH polarization configuration. The following three scattering geometries, reflection, backscattering and transmission correspond

to $\alpha = 0°$, $0° < \alpha < 90°$, and $\alpha = 90°$, respectively. The components of the stiffness elastic tensor are determined by representing the sound velocities (Figure S8) by the Christoffel's equation:

$$c_{\text{Q-L}}(\alpha) = \sqrt{\frac{-A_1+\sqrt{A_1^2-4A_2}}{2\rho}}, \tag{S2}$$

$$c_{\text{Q-T}}(\alpha) = \sqrt{\frac{-A_1-\sqrt{A_1^2-4A_2}}{2\rho}}, \tag{S3}$$

$$c_{\text{P-T}}(\alpha) = \sqrt{\frac{A_3}{\rho}}, \tag{S4}$$

where

$$A_1 = -(C_{11}\sin^2\alpha + C_{33}\cos^2\alpha + C_{44}), \tag{S5}$$

$$A_2 = C_{11}C_{44}\sin^4\alpha + \sin^2\alpha\cos^2\alpha(C_{11}C_{33} - \mathrm{C}_{13}^2 - 2C_{13}C_{44}) + C_{33}C_{44}\cos^4\alpha, \tag{S6}$$

$$A_2 = C_{66}\sin^2\alpha + C_{44}\cos^2\alpha, \tag{S7}$$

ρ is the mass density.

Based on the BLS-measured, direction-dependent sound velocities, nonlinear χ^2 fitting was conducted to obtain the elastic stiffness constants. The χ^2 is defined as:

$$\chi^2 = \sum_i \frac{[c_{i,\text{fit}}(C_{11}, C_{13}, C_{33}, C_{44}, C_{66},\alpha) - c_{i,\text{exp}}(\alpha)]^2}{(\Delta c_{i,\text{exp}})^2}, \tag{S8}$$

where: $c_{\text{i,fit}}$ and $c_{\text{i,exp}}$ are the fitted and experimental sound velocities, respectively, $\Delta c_{\text{i,exp}}$ is the uncertainty of the measured sound velocity,[7] and the summation is over all experimental sound velocities.

The use of the independent constants of the elastic tensor is not limited to the theoretical representation of the sound velocities $c_{\text{Q-L}}$, $c_{\text{Q-T}}$ and $c_{\text{P-T}}$. They were used to calculate the engineering mechanical properties,[6] including the in-plane and cross-plane Young's moduli ($E_{\parallel}$, $E_{\perp}$), shear moduli (G_{12}, G_{13}), and Poisson's ratios (ν_{31}, ν_{12}).

Supporting figures and tables for sections S3 and S4.

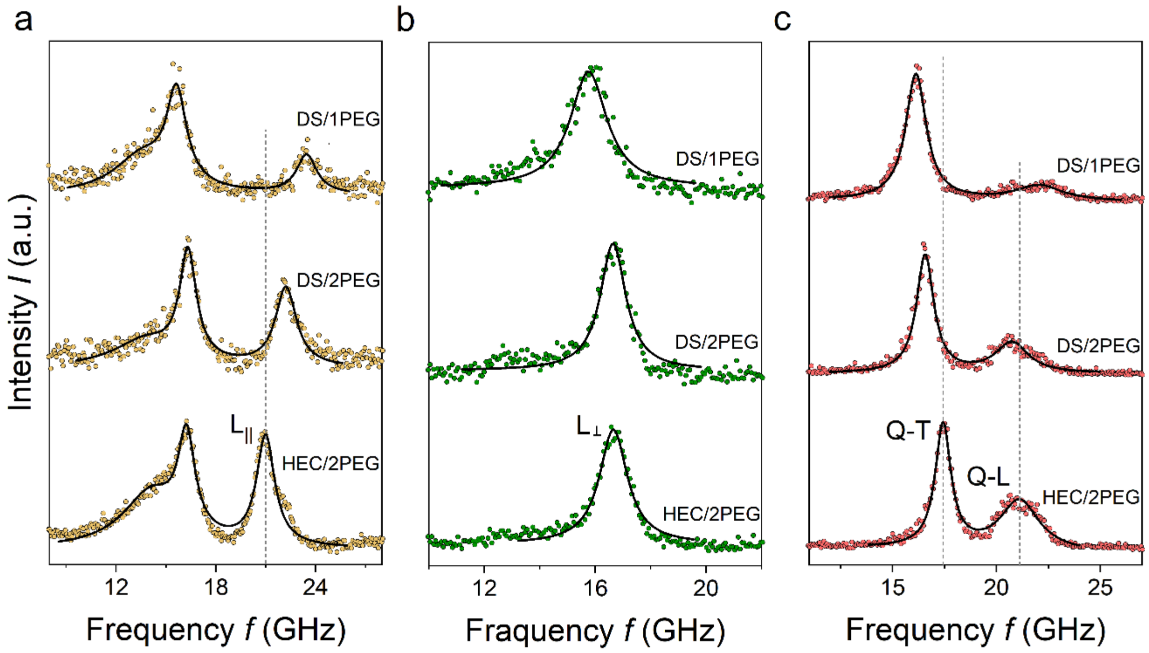

Figure S6. Experimental VV BLS spectra recorded in a) transmission, b) reflection, and c) backscattering geometries at an incident angle $\beta = 45°$. The top spectra are for DS/1PEG with 82 vol% Hec content. The middle spectra are for DS/2PEG with 70 vol% Hec content. The bottom spectra are for Hec/2PEG with 54 vol% Hec content.

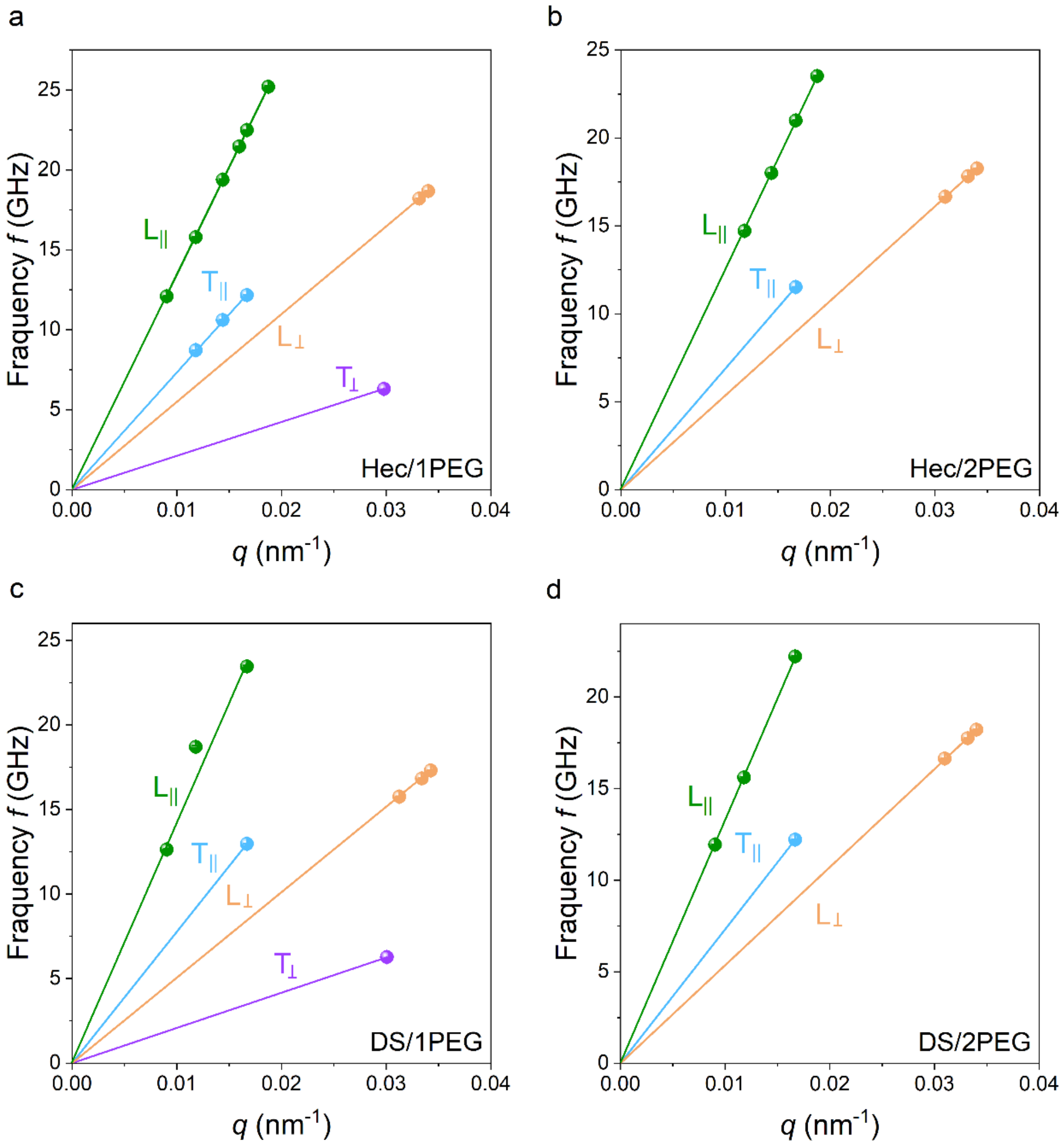

Figure S7. Dispersion relation f (q) for acoustic modes of a) Hec/1PEG, b) Hec/2PEG, c) DS/1PEG, and d) DS/2PEG recorded at the transmission geometry in VV ($L_{\parallel}$) and VH ($T_{\parallel}$) polarization configurations and at the reflection geometry in VV ($L_{\perp}$) and VH ($T_{\perp}$) polarization configurations. For the representation of the data in the reflection geometry for Hec/2PEG, $n = 1.50$ was used. For the other samples, $n = 1.49$ was used. Because of the very weak VH signal in the reflection geometry, we were unable to measure in this configuration for the Hec/2PEG and DS/2PEG samples.

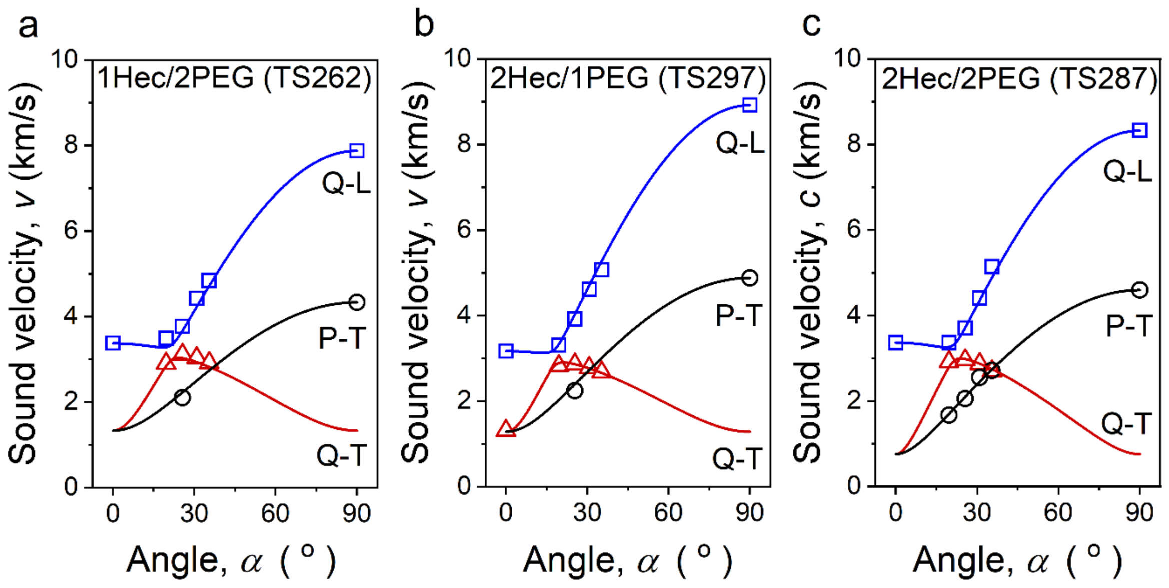

Figure S8. Experimental direction-dependent sound velocities (symbols) with theoretical representations (lines) for a) Hec/2PEG, b) DS/1PEG, and c) DS/2PEG.

Table S3. Summary of elastic stiffness constants. Composition (Hec vol%, Hec wt%), density (ρ), refractive index (n), and elastic stiffness constants (C_{11}, C_{12}, C_{13}, C_{33}, C_{44}, and C_{66}) of the Hec/PEG hybrid Bragg stack films. The results are based on fitting with two free parameters. The uncertainties are computed by assuming that the sound velocities have a relative uncertainty of 2%.

Sample ID	Hec vol%	Hec wt%	ρ (g/cm^3)	n	C_{11} (GPa)	C_{12} (GPa)	C_{13} (GPa)	C_{33} (GPa)	C_{44} (GPa)	C_{66} (GPa)
Hec/2PEG	54	73	2.03	1.49	125.9 ± 3.0	49.7 ± 4.2	3.0 ± 1.7	23.1 ± 0.5	3.6 ± 0.5	38.1 ± 1.5
Hec/1PEG	70	84	2.26	1.49	161.1 ± 3.2	65.3 ± 4.0	2.9 ± 2.2	26.9 ± 0.5	3.4 ± 0.1	47.9 ± 1.2
DS/2PEG	70	84	2.26	1.49	156.6 ± 3.6	61.2 ± 4.8	6.7 ± 1.8	25.6 ± 0.5	1.6 ± 0.6	47.7 ± 1.6
DS/1PEG	82	91	2.43	1.50	193.3 ± 4.1	77.5 ± 5.5	5.5 ± 2.5	24.4 ± 0.6	4.0 ± 0.2	57.9 ± 1.8

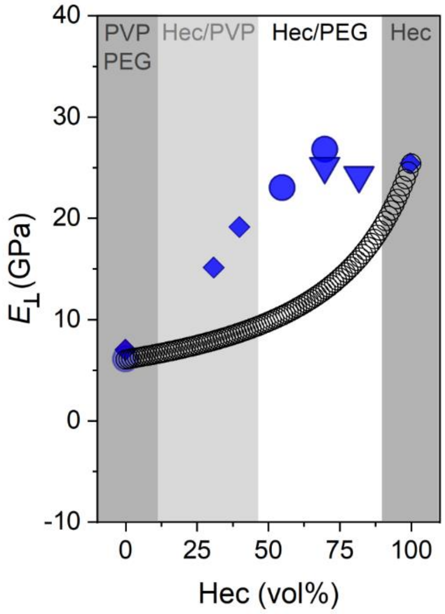

Figure S9. Composition dependence of The experimental cross-plane Young's modulus ($E_\perp$, blue symbols) plotted vs. the Hec vol%. The open symbols denote the computed Young's modulus predicted from the Wood's law.

S5. Thermal measurements

The in-plane thermal diffusivity was determined by lock-in thermography, and the cross-plane thermal conductivity by the photoacoustic method. For the determination of the in-plane thermal conductivity, the density and the specific heat are needed. Therefore, Helium pycnometry and differential scanning calorimetry (DSC) were used.

Helium pycnometry

The density of the samples was measured by helium pycnometry. Therefore, an Ultrapyc 1200e (Quantachrome Instruments) was used. Prior to each measurement the volume of the empty measurement cell was measured. Afterwards, small pieces of the free-standing films were weighed into the sample cell with a nominal volume of 1.8 cm^3. One hundred runs were performed to determine the volume of the films at room temperature. By knowing the mass (measured on a fine balance) and the volume, the density of the samples was calculated.

Differential scanning calorimetry

The specific heat capacity was determined by DSC measurements according to the ASTM E1269 standard. The samples were freeze ground for better processability and contact to the DSC pans. The measurements were performed on a TA instruments Discovery DSC 2500. The

temperature profile ranged from -40 to 200 °C using a heating rate of 20 K min^{-1} with a nitrogen flow of 50 mL min^{-1}. For all calculations the specific heat capacity at 25 °C was used.

Lock-in infrared thermography

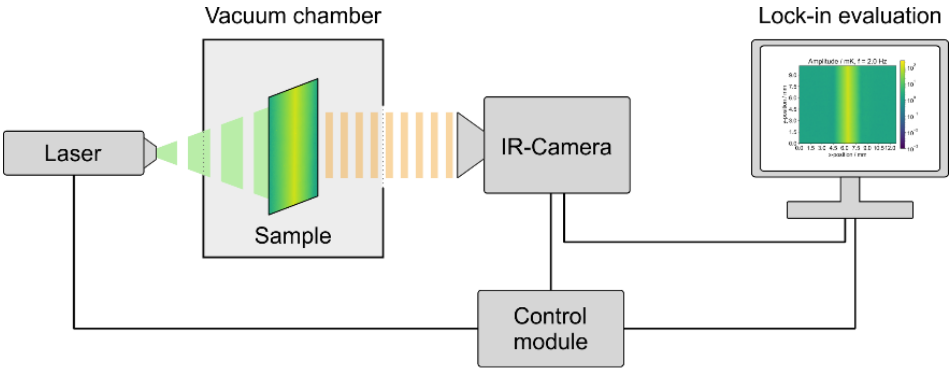

Figure S10. Schematic of the Lock-in infrared thermography measurement setup and online data evaluation.

Thermal diffusivity measurements are conducted using Lock-in infrared thermography (LIT, Figure S10). Samples are coated with 20 nm of carbon on either side and mounted on a sample holder. The holder is set in a vacuum chamber with an optically transparent window behind and an IR-transparent sapphire window in front of the sample. This allows measurements at approximately 0.01 mbar and minimizes heat losses to the environment, which would cause an overestimation of the thermal diffusivity. An intensity modulated line laser (Schäfter+Kirchhoff, λ=520 nm, P_0=55 mW) is focused on the back side of the sample. This causes temperature modulations inside the free-standing sample that are monitored with an Infratec ImageIR 9430 research IR-camera (spectral window: 2.0 – 5.5 µm) mounted with an M=1.0x microscopy objective. Measurements are conducted at an excitation frequency of 2.0 Hz, a duty cycle of 50%, camera frame rate of 20 Hz and over the course of 200 periods. Online lock-in evaluation using the IRBIS active online software directly provides phase and amplitude images (Figure S11). Linearization of both signals and fitting perpendicular to the laser line allows calculation of the thermal diffusivity by means of the slope method for thermally thin films.

From the linearized data the slope (m) of both amplitude (T) and phase data (φ) are obtained. The in-plane thermal diffusivity ($\alpha_{||}$) can then be calculated according to:

$$m_{\Phi} \cdot m_{\ln(T)} = \frac{\pi f_{lock-in}}{\alpha_{||}} \tag{S9}$$

Together with the density (ρ), determined via helium pycnometry, and the specific heat capacity (c_p), determined via DSC, the in-plane thermal conductivity can be calculated as:

$$\kappa_{||} = \alpha_{||} \cdot c_p \cdot \rho \tag{S10}$$

Two films are measured for each composition.

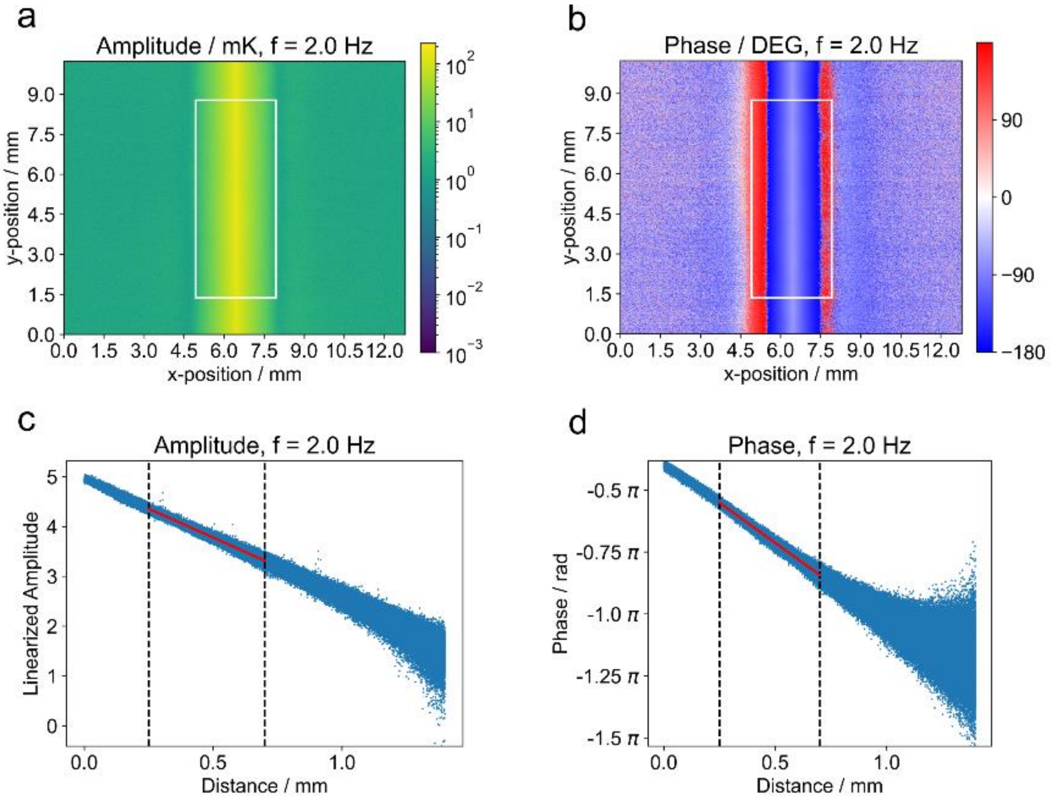

Figure S11. Typical analysis procedure of LIT. The raw Amplitude a) and Phase data b) are evaluated perpendicular to the laser-line inside the area shown by the white box. The respective 2D plots with linear regression are shown in c) and d).

Photoacoustic method

The cross-plane thermal conductivity was determined by the photoacoustic method. The thermal properties are determined by relating a measured pressure signal to the sample surface temperature and fitting the phase shift to a multilayer model. Therefore, a modulated laser beam (λ = 488 nm) periodically heats the sample. For good absorption of the laser energy, a thin Au transducer layer (~100 nm) was coated on the sample surface. For photoacoustic characterization, the samples were spray-coated on a glass substrate. The layout of the measurement cell above the sample is shown in Figure S12. The gas tight cell is filled with a helium pressure of 20 psi. The microphone (Bruel&Kjaer, 4398-A-011) connected to the cell measures the pressure signal, which is induced by the periodic heat conduction from the sample surface to the gas phase. As shown in Figure S12, the microphone is linked to a lock-in amplifier with integrated signal generator (Zurich instruments, HF2LI). The signal generator feeds the

electro-optic modulator (EOM, Conoptics, M25A) with a sinusoidal signal, and therefore the frequency of the modulated laser beam can be swept.

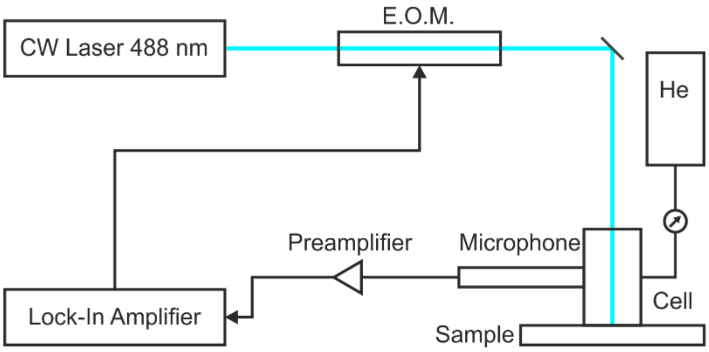

Figure S12. Schematic setup with a continuous wave laser being passed through an EOM to provide the modulated heat source. The photoacoustic signal is measured using a microphone in the pressurized cell.[8]

The phase shift is detected as a function of the frequency in a range from 310 to 9210 Hz. The signal is normalized with the phase shift signal of a thermally thick glass sample (1 mm) with known thermal properties, to determine the setups transfer function. Fitting was done according to the generalized multilayer model of Hu et al.[9] assuming one-dimensional heat transfer. Contact resistances are not taken into account. The thickness of the samples was determined by confocal microscopy. Input parameters are the thermal properties of the quartz substrate and the Au transducer layer. While the transducer layer thickness is needed, the substrate is assumed to be thermally thick. Uncertainties in sample and transducer thickness are taken into account by using a Monte-Carlo approach. In doing so, three independent measurements are analyzed using 1000 iterations with randomly selected sample and transducer thickness based on their Gaussian distribution. Afterwards, the errors due to uncertainty in thickness and the errors due to fitting, based on the Jacobian matrices and the mean square error, were added.[8] Still, all errors were assumed to be Gaussian distributed, and the $\pm 1/e$ confidence intervals are used as error bars. For every composition three samples were measured and averaged.

References

1. Breu, J.; Seidl, W.; Stoll, A. J.; Lange, K. G.; Probst, T. U., Charge Homogeneity in Synthetic Fluorohectorite. *Chem. Mater.* **2001,** *13* (11), 4213-4220.

2. Stöter, M.; Kunz, D. A.; Schmidt, M.; Hirsemann, D.; Kalo, H.; Putz, B.; Senker, J.; Breu, J., Nanoplatelets of sodium hectorite showing aspect ratios of approximately 20,000 and superior purity. *Langmuir* **2013,** *29* (4), 1280-1285.
3. Rosenfeldt, S.; Stöter, M.; Schlenk, M.; Martin, T.; Albuquerque, R. Q.; Förster, S.; Breu, J., In-Depth Insights into the Key Steps of Delamination of Charged 2D Nanomaterials. *Langmuir* **2016,** *32* (41), 10582-10588.
4. Stöter, M.; Gödrich, S.; Feicht, P.; Rosenfeldt, S.; Thurn, H.; Neubauer, J. W.; Seuss, M.; Lindner, P.; Kalo, H.; Möller, M.; Fery, A.; Förster, S.; Papastavrou, G.; Breu, J., Controlled Exfoliation of Layered Silicate Heterostructures into Bilayers and Their Conversion into Giant Janus Platelets. *Angew. Chem. Int. Ed.* **2016,** *55* (26), 7398-7402.
5. Stempflé, P.; Pantalé, O.; Rousseau, M.; Lopez, E.; Bourrat, X., Mechanical properties of the elemental nanocomponents of nacre structure. *Mater. Sci. Eng. C* **2010,** *30* (5), 715-721.
6. Cusack, S.; Miller, A., Determination of the elastic constants of collagen by Brillouin light scattering. *J. Mol. Biol.* **1979,** *135* (1), 39-51.
7. Zgonik, M.; Bernasconi, P.; Duelli, M.; Schlesser, R.; Günter, P.; Garrett, M. H.; Rytz, D.; Zhu, Y.; Wu, X., Dielectric, elastic, piezoelectric, electro-optic, and elasto-optic tensors of $BaTiO_3$ crystals. *Phys. Rev. B* **1994,** *50* (9), 5941-5949.
8. Herrmann, K.; Pech-May, N. W.; Retsch, M., Photoacoustic thermal characterization of low thermal diffusivity thin films. *Photoacoustics* **2021,** *22,* 100246.
9. Hu, H.; Wang, X.; Xu, X., Generalized theory of the photoacoustic effect in a multilayer material. *J. Appl. Phys.* **1999,** *86* (7), 3953-3958.
